# A Checklist of the Ornamental Vascular Flora of Sicily

**DOI:** 10.3390/plants14050795

**Published:** 2025-03-04

**Authors:** Emilio Di Gristina, Giulio Barone, Gianniantonio Domina, Emilio Badalamenti, Maria Letizia Gargano, Giuseppe Venturella, Raimondo Pardi

**Affiliations:** 1 Department of Agricultural, Food and Forest Sciences, University of Palermo, Viale delle Scienze, Bldg. 4, 90128 Palermo, Italy; emilio.digristina@unipa.it (E.D.G.); gianniantonio.domina@unipa.it (G.D.); emilio.badalamenti@unipa.it (E.B.); giuseppe.venturella@unipa.it (G.V.); raimondo.pardi@unipa.it (R.P.); 2 NBFC—National Biodiversity Future Centre, 90133 Palermo, Italy; 3 Department of Soil, Plant and Food Sciences, University of Bari Aldo Moro, Via G. Amendola, 165/A, 70126 Bari, Italy; marialetizia.gargano@uniba.it

**Keywords:** alien species, historic villas, parks, private gardens, street trees, urban greenery, urban biodiversity

## Abstract

Based on literature data and new field investigations, a checklist of the ornamental vascular flora of Sicily is presented. Trees, shrubs, herbaceous, and succulent plants growing in street trees, parks, private gardens, and historic villas of Sicily are included in the checklist. For each taxon, information on growth forms, geographical and biome origin, resident time, and status (native/alien) in Italy is provided. A total of 928 taxa (including 823 species s. str., 33 cultivars, 31 hybrids, 24 varieties, 9 subspecies, and 8 forms), belonging to 486 genera included in 138 families, were recorded. Fabaceae, Rosaceae, Asteraceae, Cactaceae, Asparagaceae, Arecaceae, and Malvaceae are the richest families in taxa. Phanerophytes are the prevalent growth form, and the main part of such flora consists of Asiatic and American taxa. Taxa from subtropical and tropical biomes showed a prevalent presence; this is a peculiarity which characterizes the Sicilian ornamental flora. *Delonix regia* emerges as a peculiar case, representing the only documented cases of open-air cultivation in both Sicily and the entire country. A significant percentage of taxa (41%) is included in the list of alien vascular flora of Italy, showing a strong predominance of casual and naturalized neophytes alien species.

## 1. Introduction

Currently, rapid urbanization, climate change, pollution, water shortages, and the decline of human well-being are challenges that cities need to address [1]. To deal with such challenges, urban ecosystems must enhance the environmental services offered through green urban infrastructure. Usually, ornamental plants have been used as street trees in cities and in urban parks and gardens for their aesthetic beauty. Beyond that, ornamental plants represent significant living elements in urban settings and deliver many vital ecosystem services, such as climate regulation, air, and water cleaning, nutrient recycling, the creation of productive soil, pollination, and food for animals, but also cultural benefits like recreational opportunities and the inspiration for drawings from nature [1,2]. On the other hand, many ornamental plants are toxic and can also be responsible for allergies, so in planning and managing public and private green spaces, the potential risks to human health need to be carefully evaluated [3]. Moreover, ornamental plants serve as a major source of alien plant invasions worldwide [4]. The count of alien species is consistently and gradually increasing, and the occurrence of biological invasions is recognized to harm biodiversity, ecosystem services, human health, and the economy [5,6]. In this context, the case of *Ailanthus altissima* (Mill.) Swingle is a very common example. Indeed, it is an exotic ornamental tree which represents a significant management challenge as it is both an invasive and allergenic species [7]. Recent scientific studies highlighted the importance of recognizing the existence of alien species and the potential interactions they may have with native flora. The combined strategy for the management of non-native plant species highlights the need for multidisciplinary approaches to reduce adverse effects and promote a sustainable coexistence between native and alien species [8]. Sicily, the largest island in the Mediterranean Basin, well known for its native and endemic flora, hosts a significant number of exotic ornamental plants. Since the Sicilian territory includes 11 bioclimatic zones ranging from the Infra-Mediterranean to the upper Cryo-Mediterranean [9], the island can host taxa from different climates. The ornamental flora of Sicily has been investigated for over forty years. Several studies have dealt with the census of plants in historic parks and gardens, focusing attention not only on their taxonomic, chorological, and ecological characterization but also in relation to their spatial distribution and the type of gardens. The knowledge of this plant heritage, which for its characteristics has no equal in other regions of Italy, is of undoubted interest [10]. A good part of the contributions published to date concern the ornamental flora of the city of Palermo, which is the Sicilian city with the largest number of historic parks and gardens. This is certainly due to the role of capital that the city itself had in the past; it was here that lived most of the members of the sumptuous nobility and wealthy Sicilian bourgeoisie who, especially from the beginning of the 19th century, began to enrich their villas with new plants inspired and certainly helped by the activities of the Palermo Botanical Garden. The study of Sicilian ornamental flora has not only concerned Palermo and its province but also other urban areas of western Sicily (Trapani and Agrigento) and eastern Sicily (Messina, Catania, Siracusa, and Ragusa) [10]. Although an exhaustive inventory of ornamental taxa has recently been produced for Apulia (southern Italy) [7], in other regions of Italy previous studies have been little concerned by the ornamental flora whereas many references addressing, more broadly, allochthonous or alien species can be found in publications and books [11]. In this survey, we report a first contribution on the checklist of Sicilian ornamental vascular flora. To this end, the different floristic lists published so far have been gathered together and the literature data have been integrated with a large number of censuses conducted throughout the Sicilian territory.

## 2. Results

**Figure 1:**
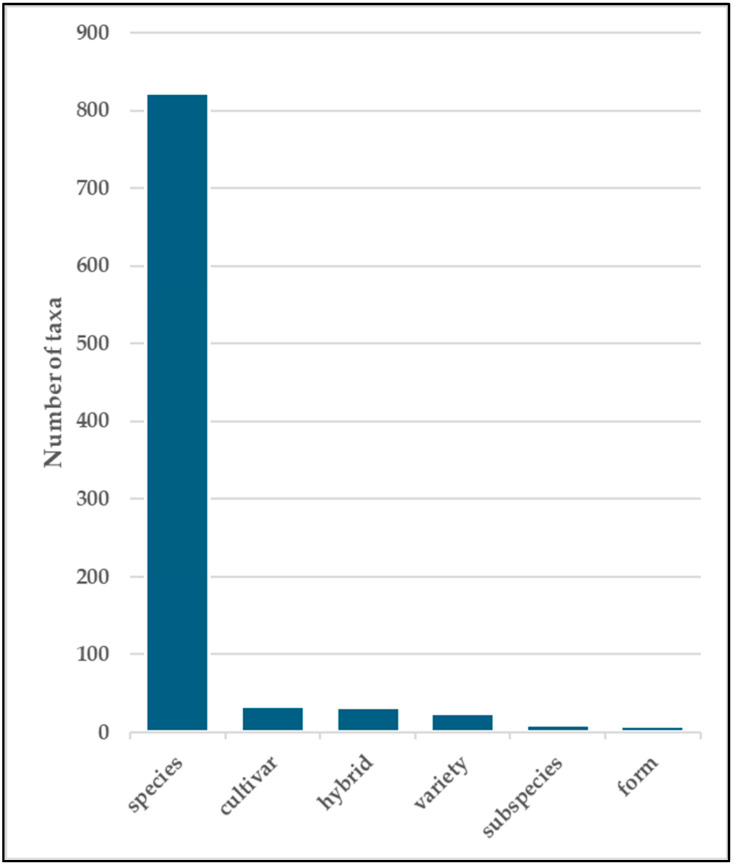
Taxonomic ranks of ornamental taxa.

**Figure 2:**
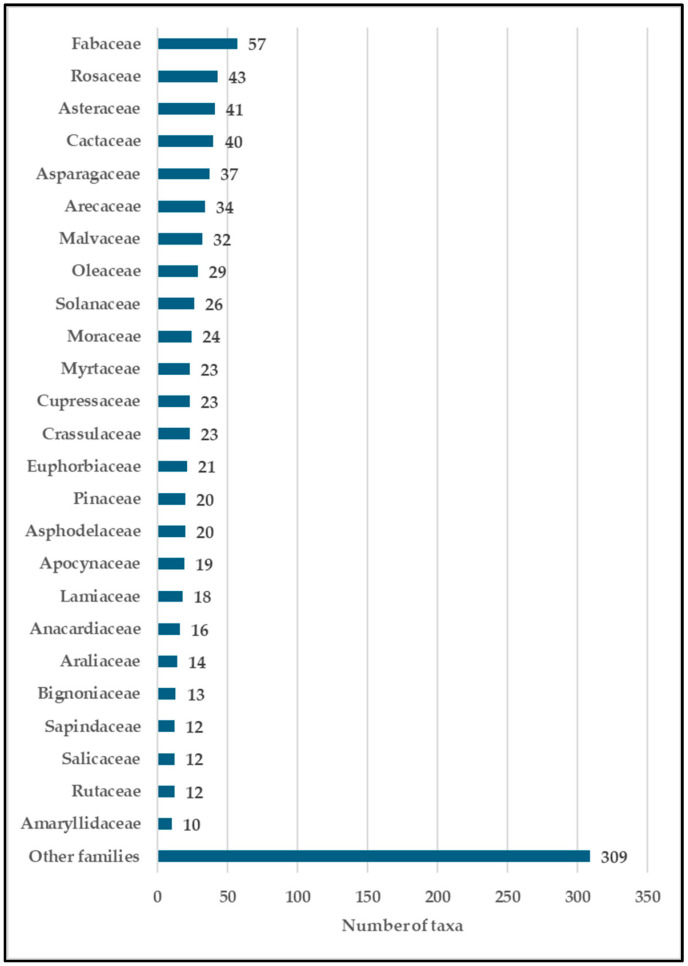
The number of taxa per families in the ornamental flora of Sicily.

A total of 928 taxa, including 823 species s. str., 33 cultivar, 31 hybrids, 24 varieties, 9 subspecies, and 8 forms, are listed (Figure 1). The recorded taxa belong to 486 genera of 138 families (Table S1). A total of 8 taxa belong to the Pteridophyta, 61 to the Pinophyta, 5 to the Cycadophyta, 1 to the Ginkgophyta, and 853 to the Magnoliophyta (693 Magnoliopsida and 160 Liliopsida). The families with the highest number of specific and subspecific taxa are the following: Fabaceae (57 taxa, 6.1%), Rosaceae (43 taxa, 4.6%), Asteraceae (41 taxa, 4.4%), Cactaceae (40 taxa, 4.3%), Asparagaceae (37 taxa, 4.0%), Arecaceae (34 taxa, 3.7%), Malvaceae (32 taxa, 3.4%), Oleaceae (29 taxa, 3.1%), Solanaceae (26 taxa, 2.8%), Moraceae (24, taxa, 2.6%), Cupressaceae, Crassulaceae, and Myrtaceae (23 taxa, 2.5%), Euphorbiaceae (21 taxa, 2.3%), Pinaceae, and Asphodelaceae (20 taxa, 2.2%) (Figure 2).

The most representative genera are *Euphorbia* and *Ficus* (19 taxa), *Opuntia* (14 taxa), *Aloe* (11 taxa), *Agave* and *Eucalyptus* (10 taxa), *Citrus*, *Pinus*, and *Prunus* (9 taxa), *Jasminum*, and *Ligustrum* (8 taxa).

**Figure 3:**
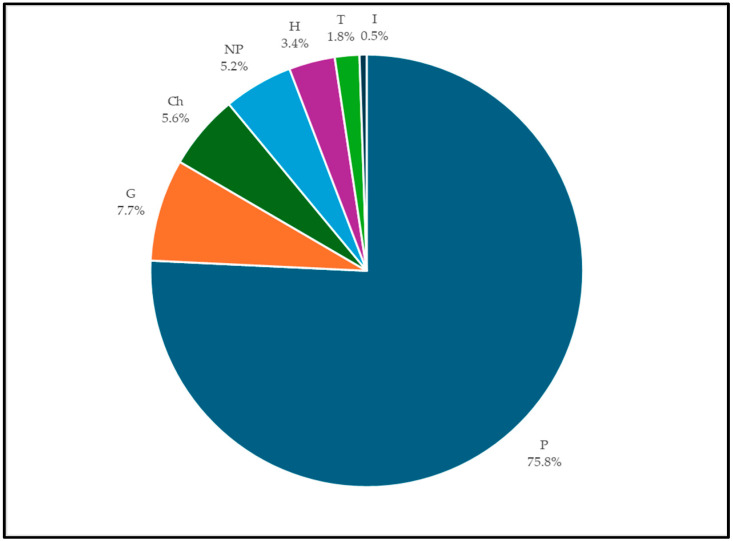
A growth form spectrum of ornamental taxa categorized according to [12,13] (P, Phanerophytes; NP, Nanophanerophytes; Ch, Chamaephytes; H, Hemicryptophytes; G, Geophytes; T, Therophytes; and I, Idrophytes).

The growth form spectrum (Figure 3) shows a clear prevalence of phanerophytes (in particular the scapose and caespitose) which overall reach 703 taxa, accounting for 75.8%; geophytes (71 taxa, 7.7%), chamaephytes (52 taxa, 5.6%), nanophanerophytes (48 taxa, 5.2%), and hemicryptophytes (32 taxa, 3.4%). Lower percentage values are shown by therophytes (17 taxa, 1.8%) and hydrophytes (5 taxa, 0.5%).

**Figure 4:**
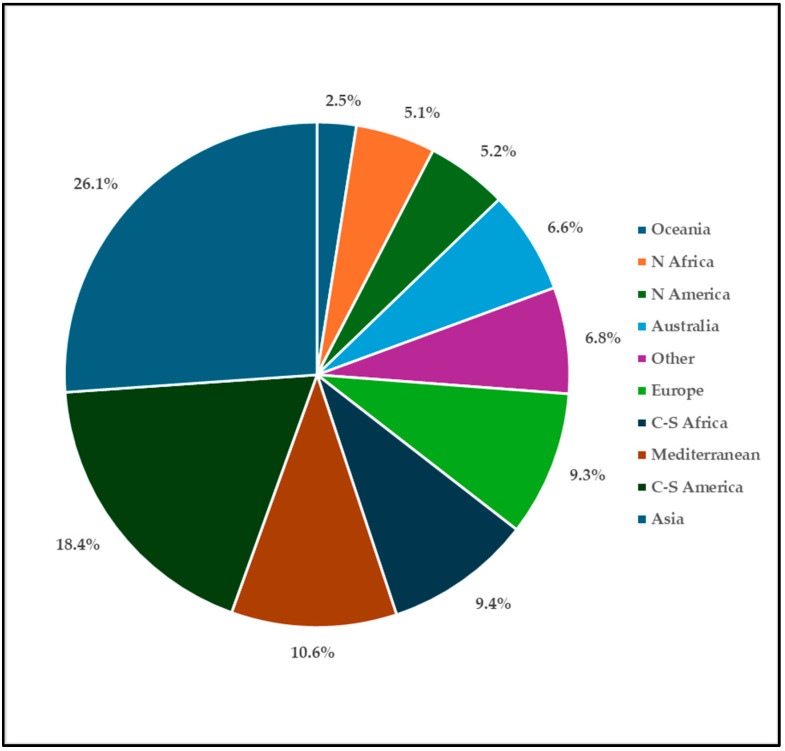
The percentage of taxa per geographical origin (according [12]).

The above percentage breakdown is due to the fact that trees, shrubs, and geophytes demand less cultivation care than other types of plants. Annuals, specifically, thrive primarily in private gardens, where the owners’ care and commitment are more significant. Likewise, aquatic plants are rarely seen, primarily because of the limited water resources and the high costs associated with their maintenance [3]. Regarding the geographical origin (Figure 4), the contingents with the greatest number of representatives are the Asian (26.1%), the American (5.2% North, and 18.4% Central and South America), and the African (14.5% of which 9.4% are from Southern Africa).

**Figure 5:**
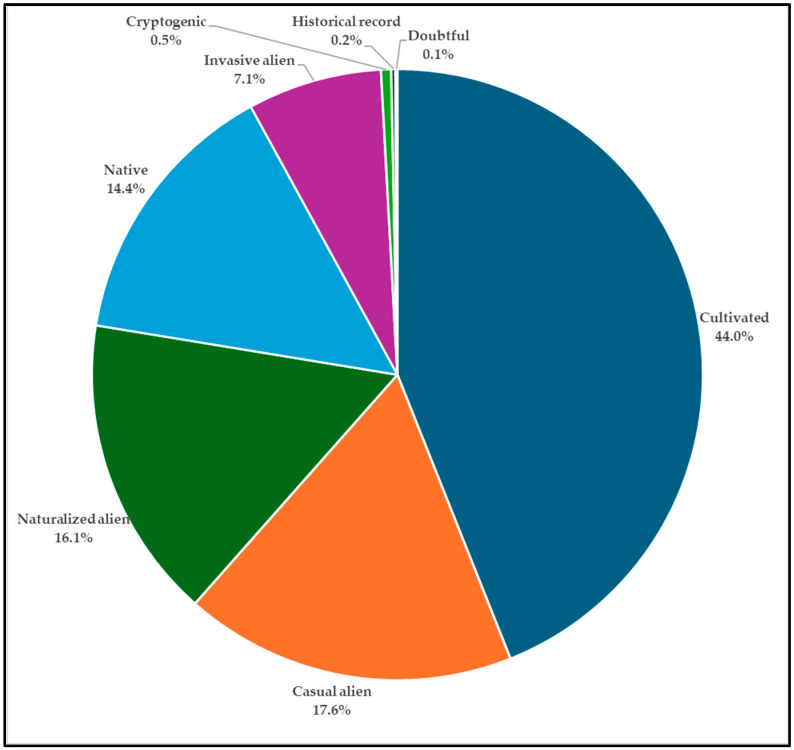
Status in Italy (according to [14,15]) and the percentage of ornamental taxa.

The Mediterranean contingent amounts to 10.6%, demonstrating a certain interest also in the use of native plants for decorative purposes. The flora of the European continent accounts to 9.3% and that of Oceania to 9.1% (mainly from Australia, 6.6%). Finally, 6.8% is represented by taxa of different origins: Pacific Islands, Atlantic Islands (Canary Islands, Azores), horticultural, and artificial hybrids. The most represented origin biome is the temperate (321 taxa, 34.6%); however, adding the data of the subtropical and tropical biomes (in total 536 taxa, 57.7%), most of the taxa come from warm climates. Only 134 taxa (14.0%) are native to Italy, while 786 taxa (85.0%) are allochthonous; the remaining eight taxa (1.0%) are included in the cryptogenic, historical record, and doubtful categories. Concerning the tendency of naturalization on the allochthonous taxa, 408 taxa (44.0%) are cultivated, 163 taxa (18.0%) are casual alien (i.e., they exhibit a trend towards naturalization yet do not form populations separate from the cultivated plants), 149 taxa (16.0%) are naturalized (i.e., they tend to form stable populations) and 66 taxa (7.0%) are invasive alien (i.e., they could pose a threat to biodiversity by competing with native species) (Figure 5).

## 3. Discussion

**Figure 6:**
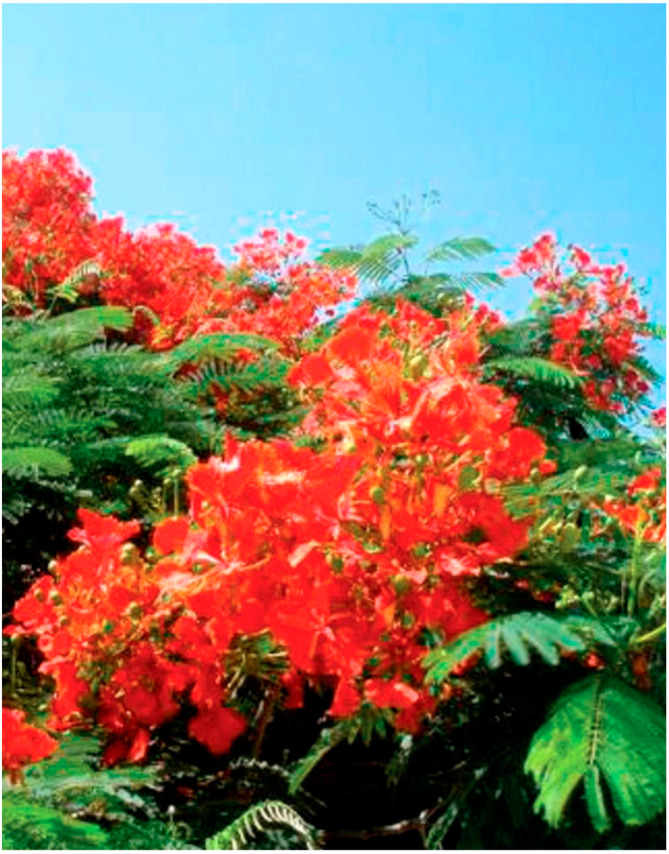
Blooms of the *Delonix regia* in the urban center of Donnalucata (Ragusa, SE Sicily).

Since the Sicilian territory includes a wide range of bioclimatic areas [9], the island can host taxa originating from different climates. Indeed, in the mountainous or hilly areas (Madonie, Nebrodi, province of Enna, Etna volcano, Iblean Plateau, and Mounts Sicani) there are typical taxa from cooler climates such as *Abies alba* Mill., *A. cephalonica* Loudon, *A*. *nebrodensis* (Lojac.) Mattei, *A*. *pinsapo* Boiss., *Amelanchier laevis* Wiegand, *Cedrus atlantica* (Endl.) Manetti ex Carrière, *Cedrus deodara* (Roxb. ex D. Don) G. Don, *C*. *libani* A. Rich., *Picea abies* (L.) H. Karst., *P*. *pungens* Engelm., *P*. *smithiana* (Willd.) Boiss., *Sequoia sempervirens* (D. Don) Endl., and *Taxus baccata* L.. As for *Abies nebrodensis*, it is an endemic species with limited distribution on the Madonie Mountains and at risk of extinction. Among the ex situ conservation actions proposed within LIFE projects (LIFE Natura and LIFE4FIR) the adoption of plants by local populations has been foreseen. This proposal, which was favorably received, has led to the widespread use of *A*. *nebrodensis* as an ornamental plant in recent times. Along the coasts of Sicily, where the climate is warmer, taxa of the subtropical and tropical flora prevail. This is certainly the floristic component that peculiarly characterizes the Sicilian ornamental flora. In fact, these taxa, planted in open air, manage to flower and bear fruit abundantly, arousing the wonder of foreigners visiting the island. Furthermore, several of these taxa, which in Sicily grow vigorously and luxuriantly, in the rest of Italy and Europe either do not survive or live with difficulty and rarely bloom. Under these conditions, lush gardens and magnificent villas arose in the coastal Sicilian cities, of such splendor and richness as to evoke an almost phantasmagorical impression of the tropics in the heart of the Mediterranean. In this sense, there are several taxa of high ornamental value, not only for the beauty of the flowering but also for the vegetative habitus, which now characterize the Sicilian coastal urban greenery. The survey of the Sicilian ornamental heritage revealed the presence of rare taxa both for the Sicilian context and for the Italian and Mediterranean one. Some of them are also relevant for their size, age, historical value, the role they play in the characterization of individual plants, and of relative contexts. These taxa, therefore, constitute real floristic and cultural peculiarities. The most significant ones are reported below as follows: *Acokanthera oppositifolia* (Lam.) Codd, *Agathis robusta* (C. Moore ex F. Muell.) F. M.Bailey, *Allocasuarina verticillata* (Lam.) L. A. S.Johnson, *Aloidendron barberae* (Dyer) Klopper and Gideon F. Sm., *Alpinia zerumbet* (Pers.) B. L. Burtt and R. M. Sm., *Araucaria araucana* (Molina) K. Koch, *A*. *bidwillii* Hook., *A*. *cunninghamii* Mudie, *A*. *rulei* F. Muell., *Bosea yervamora* L., *Brahea calcarea* Liebm., *B*. *dulcis* (Kunth) Mart., *Butia yatay* (Mart.) Becc., *Calycanthus floridus* L., *Camphora officinarum* Boerh. ex Fabr., *Ceratozamia mexicana* Brongn., *Combretum indicum* (L.) De Filipps, *Cordia francisci* Ten., *C*. *myxa* L., *Corynocarpus laevigatus* J. R. Forst. and G. Forst., *Decaisnea fargesii* Franch., *Delonix regia* (Bojer) Raf., *Dracaena draco* L., *Ehretia tinifolia* L., *Elaeodendron australe* Vent., *Enterolobium contortisiliquum* (Vell.) Morong, *Eriocephalus africanus* L., *Erythrina afra* Thunb., *Eucalyptus grandis* W. Hill ex Maiden, *Euphorbia candelabrum* Welw., *E*. *tirucalli* L., *E*. *triangularis* Desf. ex A. Berger, *Ficus altissima* Blume, *F*. *benghalensis* L., *F*. *benjamina* L., *F*. *elastica* Roxb. ex Hornem var. *decora* Guillaumin, *F*. *lyrata* Warb., *F*. *macrophylla* Pers. f. *columnaris* (C. Moore) D. J. Dixon, *F*. *macrophylla* f. *whitakeri* Raimondo and Bajona, *F*. *racemosa* L., *F*. *virens* Aiton, *Fontanesia philliraeoides* Labill., *Gardenia thunbergia* Thunb., *Halleria lucida* L., *Howea belmoreana* (C. Moore and F. Muell.) Becc., *Ipomoea alba* L., *Jacaranda mimosifolia* D. Don, *Justicia adhatoda* L., *J*. *spicigera* Schltdl., *Kleinia neriifolia* Haw., *Laurus azorica* (Seub.) Franco, *Livistona decora* (W. Bull) Dowe, *Mandevilla laxa* (Ruiz and Pav.) Woodson, *Nolina parviflora* (Kunth) Hemsl., *Opuntia streptacantha* Lem., *O*. *tomentosa* Salm-Dyck, *Oreopanax nymphaeifolius* (Hibberd) Decne. and Planch. ex G. Nicholson, *Osteomeles schweriniae* C. K. Schneid., *Pararchidendron pruinosum* (Benth.) I. C. Nielsen, *Phoenix loureiroi* Kunth, *P*. *rupicola* T. Anderson, *Photinia davidiana* (Decne.) Cardot, *Picea pungens* Engelm. ‘Glauca’, *P*. *smithiana* (Willd.) Boiss., *Pisoniella arborescens* (Lag. and Rodr.) Standl., *Pittosporum* undulatum Vent., *Plumeria rubra* L., *Podocarpus neriifolius* D. Don, *Portulacaria afra* Jacq., *Puya* ×*berteroniana* Mez, *Quercus polymorpha* Schltdl. and Cham., *Searsia lancea* (L. f.) F. A. Barkley, *Sequoia sempervirens* (D. Don) Endl., *Sparrmannia africana* L. f., *Thuja plicata* Donn ex D. Don, *Thunbergia coccinea* Wall. ex D. Don, *Xanthoceras sorbifolium* Bunge, *X*. *preissii* Endl., *Yucca faxoniana* (Trel.) Sarg., and *Zamia furfuracea* L. f. ex Aiton. Among the above-mentioned taxa, *Delonix regia*, *Ficus macrophylla* f. *columnaris*, and *Plumeria rubra* deserve a special mention. *Delonix regia* is an evergreen tree native to Madagascar, among the most spectacular and ornamental in the world. It is known as ‘flamboyant’, for the showy red bloom that covers its large crown. In Sicily, an adult individual (Figure 6) capable of flowering and fruiting is found in a small private garden in the urban center of Donnalucata (Ragusa); two other young plants, not yet flowering, are found in Scoglitti and Marina di Ragusa (Ragusa). These are the only documented reports of the species being cultivated in open air conditions in both Sicily and Italy [16].

**Figure 7:**
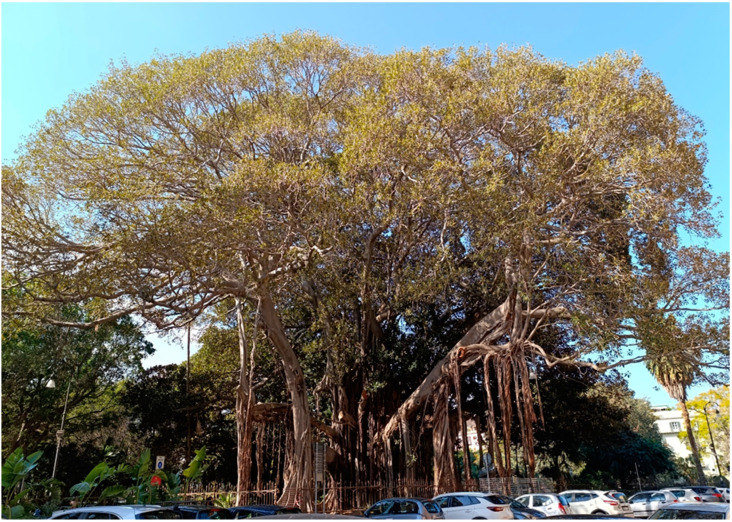
The monumental tree *Ficus macrophylla* f. *columnaris* in the Villa Garibaldi of Palermo (NW Sicily).

**Figure 8:**
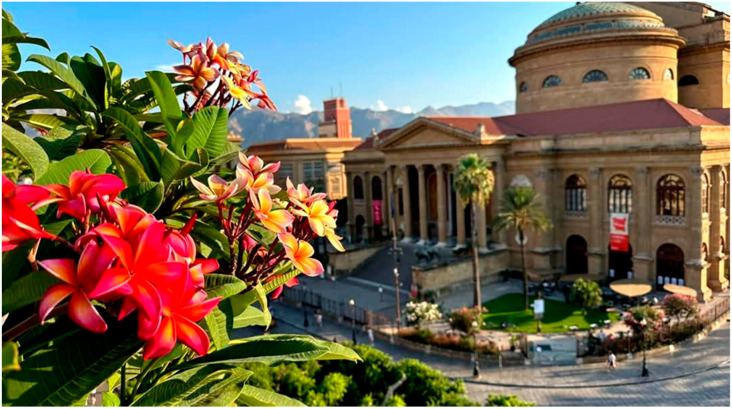
The flowering of *Plumeria rubra* in the city of Palermo.

*F*. *macrophylla* f. *columnaris* is a majestic tree native to the Lord Howe Islands (Australia), very distinctive for its particular shape. It has a large, branched crown with numerous bundles of hanging aerial roots that, once they reach the ground, grow, intertwining and joining together to form columnar roots that serve to support the large branches. In nature, it first grows as an epiphyte on other trees which it then suffocates by developing a powerful network of branches, and aerial and tabular roots to support its monumental crown. In Palermo, there are several monumental individuals, especially those of Villa Garibaldi (Figure 7) and the Botanical Garden, which are considered, for the development of the crown and volume, the largest trees in Europe [17]. *Plumeria rubra*, also known as ‘frangipani’, is a small Caribbean tree of great ornamental value due to its beautiful and fragrant flowers of different colors and forms [18]. In Hawaii, its flowers are traditionally used for the famous flower necklaces (Lei), which have become a symbol of brotherhood and hospitality. In Sicily, the species is cultivated along the coastal areas, especially in Palermo (Figure 8), where it has been renamed ‘pomelia’. Upon its introduction in Palermo, the plant was instantly valued for the stunning beauty and strong fragrance of its flowers, leading to its rapid proliferation in both private and public gardens, and its cultivation in pots on balconies and terraces, ultimately becoming a symbol of the city’s floral exhibitions. In Palermo, *P. rubra* serves not only an ornamental purpose but also holds deep cultural importance. It is a sign of fertility developed by comparison with the extraordinary strength of the plant’s vegetative power. Consequently, the white flowers are frequently utilized in the bouquets of brides in Palermo, and traditionally, the bride moving to a new home receives a plant from her mother as a gesture for family continuity [19].

Based on floristic surveys reported in the existing literature [10,20,21,22,23,24,25,26,27,28,29,30,31,32,33,34,35], our field investigations have identified 63 unpublished taxa (6.8%). Among these, the following genera *Abelia*, *Acanthus*, *Alyogyne*, *Annona*, *Archontophoenix*, *Asclepias*, *Astrophytum*, *Cochliasanthus*, *Deuterocohnia*, *Eremophila*, *Eschscholzia*, *Euryops*, *Gunnera*, *Hardenbergia*, *Harpephyllum*, *Kumara*, *Liquidambar*, *Loropetalum*, *Pachira*, *Pachypodium*, *Phlomis*, *Pyrostegia*, *Rhapis*, *Roystonea*, *Saccharum*, *Sesbania*, *Symphoricarpos*, *Talinum*, *Weigela*, and *Zamia* are reported for the first time in Sicily. These newly recorded plants contribute to further diversifying the Sicilian ornamental flora.

Common in the Sicilian gardens is the utilization of plants that offer both decorative value and productivity. Among the ornamental taxa found in this study that also have nutritional importance we should mention the following: *Annona cherimola* Mill., *Carica papaya* L., *Carya illinoinensis* (Wangenh.) K. Koch, *Casimiroa edulis* La Llave, *Citrus japonica* Thunb., *C*. *reticulata* Blanco, *C*. ×*aurantium* L., *C*. ×*aurantium* f. *aurantium*, *C*. ×*limon* (L.) Osbeck, *Corylus avellana* L., *Diospyros kaki* Thunb., *Feijoa sellowiana* (O. Berg) O. Berg, *Juglans regia* L., *Musa* ×*paradisiaca* L., *Persea americana* Mill., *Punica granatum* L., *Prunus armeniaca* L., *P. domestica* L., *P*. *dulcis* (Mill.) D. A. Webb, *P*. *persica* (L.) Batsch, *Saccharum officinarum* L., and *Vitis vinifera* L. The most important medicinal plants are *Aloe arborescens* Mill., *A. arborescens* var. *frutescens* (Salm-Dyck) Link, and *Aloe vera* L. In addition to their ornamental, productive, and medicinal roles, these taxa could be strategically used in public urban areas for educational activities, particularly in schools, where hands-on learning about plant cultivation, local biodiversity, and nutrition can be integrated into their studies. Furthermore, they hold potential for short-scale feeding initiatives aimed at children or individuals in need by providing a supplementary source of fresh produce. This approach not only enhances environmental awareness but also fosters social benefits through the intersection of aesthetics, education, and locally oriented food supplies.

On the contrary, many of the taxa found in Sicilian ornamental flora have a negative impact on human health and it is desirable to advise against their use in new gardens. The best-known ornamental genera and plants considered responsible for pollinosis and commonly used in Sicily, especially in urban areas, are *Cupressus* spp., *Hesperocyparis* spp., *Pinus* spp., *Ailanthus altissima*, *Olea europaea* L., *Quercus ilex* L., and *Populus* sp. pl. Stinging hairs inside the fruits of *Brachychiton* spp. and *Lagunaria patersonia* (Andrews) G. Don are dangerous for the mucous membranes of the eyes and mouth; in the genera *Ficus* and *Plumeria*, the latex is irritating [3]. Also, a wide number of ornamental Sicilian taxa are poisonous such as *Brugmansia arborea* (L.) Sweet, *Cascabela thevetia* (L.) Lippold, *Digitalis purpurea* L., *Drimia pancration* (Steinh.) J. C. Manning and Goldblatt, *Laburnum anagyroides* Medik., *Melia azedarach* L., *Nerium oleander* L., *Nicotiana glauca* Graham, *Ricinus communis* L., *Tagetes erecta* L., *Taxus baccata* L., and *Thuja occidentalis* L. The species selected for ornamental greenery, in principle, ought to pose no harmful effects on human health or on pets, yet attaining this outcome is challenging [3]. In fact, in many cases, decisions made by municipal administrations about ornamental plants are influenced by the visual appeal of the plants and their accessibility in garden centers. However, when planning and supervising public and private green spaces, the potential risks that plants may pose to the health of humans and pets must be considered. To this end, it would be desirable for administrators to avail themselves of the professional support of botanists to adequately guide their choices regarding urban greenery with particular reference to the health of their inhabitants [7]. Furthermore, it could be beneficial for municipal administrators to label noxious or toxic plants that are in public spaces, ensuring that both residents and visitors (especially children) are aware of the potential risks. These efforts may be complemented by educational programs at schools, where students can learn about plant identification, safe handling, and responsible interaction with greenery. In this way, communities not only minimize health hazards but also foster an informed appreciation of urban flora and the importance of biodiversity.

Finally, this survey illustrates the continuous increase of alien species in the flora of Sicily: a widespread pattern observed worldwide. The overwhelming presence (as much as 85.0%) of allochthonous ornamental taxa compared to native species is another important data point to evaluate the role of such plants used as ornamentals, especially when it comes to aliens that are potentially harmful to human health that escape cultivation and tend to form stable populations. This is the case, for example, of *Cascabela thevetia* and *Melia azedarach*, valuable ornamentals but also deadly poisonous plants that have become naturalized in Sicily. These plants, along with their seeds, are easily available at nurseries and shops. All of this should serve as an additional warning about biological invasions that continue to encounter inadequate containing measures to halt their spread. In instances where such species pose a critical threat, e.g., *Acacia saligna* (Labill.) H. L. Wendl., *Ailanthus altissima, Carpobrotus acinaciformis* (L.) L. Bolus, *C. edulis* (L.) N.E.Br., *Opuntia ficus-indica* (L.) Mill., *Parkinsonia aculeata* L., *Robinia pseudoacacia* L., and *Vachellia karroo* (Hayne) Banfi and Galasso, removal programs may be necessary. In less urgent cases, targeted management actions should be considered to limit their establishment and expansion.

Nonetheless, thanks to the financing from the National Recovery and Resilience Plan (NRRP), the issue of invasive species has re-emerged on the agenda and efforts are underway to raise awareness of the dangers associated with the introduction and spread of non-native invasive species, and to emphasize the best practices in both production and gardening to mitigate the risk [7].

To the best of our knowledge, our inventory, comprising 928 taxa, currently represents the most complete contribution to the Italian ornamental flora at the regional level. Recently, a similar approach was used to produce a comprehensive inventory for the Apulia region [7], which documented a total of 287 taxa across 179 genera and 78 families. Comparing the results of the two inventories (Sicily and Apulia), we found that 251 taxa were present in both regions, while 36 of the resulted taxa were only present in Apulia. Among these, noteworthy taxa unique to Apulia include *Cupressus cashmeriana* Royle ex Carrière, *Ficus maclellandii* King, *Pyrus calleryana* Decne., *Quercus ithaburensis* subsp. *macrolepis* (Kotschy) Hedge and Yalt., and *Quercus trojana* Webb.

## 4. Materials and Methods

Firstly, we analyzed literature data [10,20,21,22,23,24,25,26,27,28,29,30,31,32,33,34,35] on trees, shrubs, herbaceous, and succulents taxa growing in street trees, parks, private gardens, and historic villas of Sicily. We subsequently conducted new field observations in order to implement the data already available. Herbarium vouchers of the plants collected are deposited at the SAF herbarium (Department of Agricultural, Food, and Forest Science, University of Palermo). Periodic observations across the nine provinces of Sicily (Agrigento, Caltanissetta, Catania, Enna, Messina, Palermo, Ragusa, Siracusa, and Trapani) were conducted. Each province was visited twice in spring and autumn between 2022 and 2024 to assess ornamental plants. With the aim of representing the main gradients of Sicilian climate, sites located in the coastal region, in hilly areas, and in the mountain belt were visited. Each taxon was identified with the support of several floras [36,37,38,39,40,41,42,43,44]. A comparison with the living collections of the Botanical Garden of Palermo and the specimens from the Herbarium Mediterraneum Panormitanum (PAL) was also carried out. The recorded taxa are listed in Table S1. New records from Sicily are reported in Table S1 with an asterisk. The nomenclature follows the database Plants of the World Online (https://powo.science.kew.org/, accessed on 5 November 2024) [12] of the Kew Royal Botanical Gardens. The names of the taxa are listed alphabetically. In addition to species sensu stricto, the checklist also comprises forms, varieties, hybrids, and cultivars. For each taxon, the following details were noted as follows: family (according to [12]); growth form (according to [12,13]); geographical origin (derived from [12]); biome origin (according to [12]); and resident time and status (native/alien) in Italy (derived both from [14,15]). The taxa belonging to the floristic surveys from the existing literature [10,20,21,22,23,24,25,26,27,28,29,30,31,32,33,34,35] have been updated in terms of nomenclature and standardized concerning synonymy in accordance with the POWO database.

## 5. Conclusions

The topic of urban greenery is acquiring a growing interest not only from a practical point of view, but also from a scientific, not only botanical, side. In this regard, the taxonomic knowledge of plants that characterize the green spaces of cities also assumes relevance from a managerial, safety, health, and hygiene point of view. On these premises, having a wide knowledge on the nature, origin, and distribution of the plants introduced in urban spaces helps both public administrations and citizens to manage and enjoy greenery with a greater awareness. Our investigation has contributed to deepening the knowledge of the ornamental flora planted in street trees, parks, private gardens, and historic villas of the whole Sicilian region. This type of census is so far very limited at the national level. The data reported in this first contribution represents a useful tool for further investigations on the exotic component present in the ornamental greenery of the Island and on its diversity, but also for municipal administrations to guide future choices within the cities aimed at the recovery, conservation, enhancement, and qualification of the ornamental flora with particular reference to the health of the citizens and the protection of biodiversity.

## Data Availability

The data that support the findings of this study are available upon request to the corresponding author.

## Supplementary Materials

The following supporting information can be downloaded at https://www.mdpi.com/article/10.3390/plants14050795/s1; Table S1: List of the Sicilian ornamental taxa.

## Abbreviations

The following abbreviations are used in this manuscript:

POWO	Plants of the World Online database
