# Supplementary material for: A Checklist of the Ornamental Vascular Flora of Sicily
Source: Plants (Basel). 2025 Mar 4;14(5):795. doi: 10.3390/plants14050795 (PMC11901704; doi:10.3390/plants14050795)
Supplement: Supplementary file 1 [file plants-14-00795-s001.zip › plants-3499307-supplementary.pdf]

**Table S1.** List of the Sicilian ornamental taxa per Families, Growth Form (according to [41, 42]), Geographical Origin (derived from [41]), Biome Origin (according to [41]), Resident Time and Status (Native/Alien) in Italy (derived both from [43,44]). New records from Sicily are reported with an asterisk.

| FAMILIES | TAXA | GROWTH FORM | GEOGRAPHICAL ORIGIN | BIOME ORIGIN | RESIDENT TIME | STATUS IN ITALY |
|---|---|---|---|---|---|---|
| Caprifoliaceae | *Abelia ×grandiflora* (Rovelli ex André) Rehder* | P caesp | Artificial hybrid | Temperate | | Cultivated |
| Oleaceae | *Abeliophyllum distichum* Nakai | P caesp | Korea | Temperate | | Cultivated |
| Pinaceae | *Abies alba* Mill. | P scap | S Europa | Temperate | | Native |
| Pinaceae | *Abies cephalonica* Loudon | P scap | Greece | Temperate | Neophyte | Invasive alien |
| Pinaceae | *Abies nebrodensis* (Lojac.) Mattei | P scap | Sicily | Temperate | | Native |
| Pinaceae | *Abies pinsapo* Boiss. | P scap | Spain | Temperate | | Cultivated |
| Malvaceae | *Abutilon ×hybridum* Voss | P caesp | Horticultural | Subtropical | | Cultivated |
| Malvaceae | *Abutilon grandifolium* (Willd.) Sweet | P caesp | S America | Subtropical | | Cultivated |
| Malvaceae | *Abutilon theophrasti* Medik. | P caesp | C Asia | Subtropical | Archaeophyte | Invasive alien |
| Fabaceae | *Acacia dealbata* Link | P scap | Australia | Temperate | Neophyte | Invasive alien |
| Fabaceae | *Acacia melanoxylon* R.Br. | P scap | Australia | Subtropical | Neophyte | Naturalized alien |
| Fabaceae | *Acacia podalyriifolia* A.Cunn. ex G.Don | P scap | Australia | Subtropical | | Cultivated |
| Fabaceae | *Acacia retinodes* Schltdl. | P scap | Australia | Subtropical | | Cultivated |
| Fabaceae | *Acacia saligna* (Labill.) H.L.Wendl. | P scap | Australia | Subtropical | Neophyte | Invasive alien |
| Acanthaceae | *Acanthus mollis* L. 'Whitewater'* | H scap | Horticultural | Temperate | | Cultivated |
| Sapindaceae | *Acer campestre* L. | P scap | Europe, N-W Africa, W Asia | Temperate | | Native |
| Sapindaceae | *Acer campestre* L. 'Argenteo-variegatum' | P scap | Horticultural | Temperate | | Cultivated |
| Sapindaceae | *Acer negundo* L. | P scap | N-C America | Temperate | Neophyte | Invasive alien |
| Sapindaceae | *Acer platanoides* L. | P scap | Europe, C Asia | Temperate | | Native |
| Sapindaceae | *Acer pseudoplatanus* L. | P scap | Europe, Caucasus | Temperate | | Native |
| Sapindaceae | *Acer saccharinum* L. | P scap | N America | Temperate | Neophyte | Naturalized alien |
| Apocynaceae | *Acokanthera oblongifolia* (Hochst.) Benth. & Hook.f. ex B.D.Jacks. | P caesp | S Africa | Shrubland | | Cultivated |
| Apocynaceae | *Acokanthera oppositifolia* (Lam.) Codd | P caesp | S Africa | Tropical | | Cultivated |
| Actinidiaceae | *Actinidia chinensis* var. *deliciosa* (A.Chev.) A.Chev. | P lian | China | Temperate | Neophyte | Casual alien |
| Pteridaceae | *Adiantum capillus-veneris* L. | G rhiz | Cosmopolitan | Temperate | | Native |
| Crassulaceae | *Aeonium arboreum* (L.) Webb & Berthel. | NP | Canary Is. | Subtropical | Archaeophyte | Naturalized alien |
| Crassulaceae | *Aeonium arboreum* (L.) Webb & Berthel. 'Atropurpureum' | NP | Canary Is. | Subtropical | | Cultivated |
| Crassulaceae | *Aeonium arboreum* subsp. *holochrysum* (H.Y.Liu) Bañares | NP | Canary Is. | Subtropical | Archaeophyte | Naturalized alien |
| Crassulaceae | *Aeonium canariense* (L.) Webb & Berthel. | NP | Canary Is. | Subtropical | | Cultivated |
| Crassulaceae | *Aeonium decorum* Webb ex Bolle | NP | Canary Is. | Subtropical | Neophyte | Naturalized alien |
| Crassulaceae | *Aeonium gomerense* (Praeger) Praeger | NP | Canary Is. | Subtropical | Neophyte | Casual alien |
| Crassulaceae | *Aeonium lanzerottense* (Praeger) Praeger | NP | Canary Is. | Subtropical | Neophyte | Casual alien |
| Sapindaceae | *Aesculus ×carnea* Zeyh. | P scap | Artificial hybrid | Temperate | | Cultivated |

**Table S1.** List of the Sicilian ornamental taxa per Families, Growth Form (according to [41, 42]), Geographical Origin (derived from [41]), Biome Origin (according to [41]), Resident Time and Status (Native/Alien) in Italy (derived both from [43,44]). New records from Sicily are reported with an asterisk.

| FAMILIES | TAXA | GROWTH FORM | GEOGRAPHICAL ORIGIN | BIOME ORIGIN | RESIDENT TIME | STATUS IN ITALY |
|---|---|---|---|---|---|---|
| Sapindaceae | *Aesculus hippocastanum* L. | P scap | Balkan, Turkey | Temperate | Neophyte | Casual alien |
| Podocarpaceae | *Afrocarpus falcatus* (Thunb.) C.N.Page | P scap | S Africa | Subtropical | | Cultivated |
| Amaryllidaceae | *Agapanthus africanus* (L.) Hoffmanns. | G rhiz | S Africa | Subtropical | | Cultivated |
| Araucariaceae | *Agathis robusta* (C.Moore ex F.Muell.) F.M.Bailey | P scap | S-E Asia, Queensland | Tropical | | Cultivated |
| Asparagaceae | *Agave americana* L. | P caesp | Mexico | Tropical | Neophyte | Invasive alien |
| Asparagaceae | *Agave americana* L. f. luteo-marginata | P caesp | Mexico | Tropical | Neophyte | Invasive alien |
| Asparagaceae | *Agave americana* var. *marginata* Trel. | P caesp | Mexico | Tropical | Neophyte | Invasive alien |
| Asparagaceae | *Agave attenuata* Salm-Dyck | P caesp | Mexico | Subtropical | Neophyte | Naturalized alien |
| Asparagaceae | *Agave filifera* Salm-Dyck | P caesp | Mexico | Subtropical | Neophyte | Casual alien |
| Asparagaceae | *Agave horrida* Lem. ex Jacobi | P caesp | Mexico | Tropical | | Cultivated |
| Asparagaceae | *Agave salmiana* Otto ex Salm-Dyck | P caesp | Mexico | Subtropical | Neophyte | Invasive alien |
| Asparagaceae | *Agave salmiana* var. *ferox* (K.Koch) Gentry | P caesp | Mexico | Subtropical | Neophyte | Invasive alien |
| Asparagaceae | *Agave sisalana* Perrine | P caesp | Mexico | Tropical | Neophyte | Naturalized alien |
| Asparagaceae | *Agave vera-cruz* Mill. | P caesp | Mexico | Tropical | | Cultivated |
| Asteraceae | *Ageratina altissima* (L.) R.M.King & H.Rob. | P caesp, P scap | N America | Temperate | Neophyte | Naturalized alien |
| Simaroubaceae | *Ailanthus altissima* (Mill.) Swingle | P scap | China | Temperate | Neophyte | Invasive alien |
| Lardizabalaceae | *Akebia quinata* (Thunb. ex Houtt.) Decne. | P lian | China, Japan | Temperate | Neophyte | Casual alien |
| Fabaceae | *Albizia julibrissin* Durazz. | P scap | S Asia | Temperate | Neophyte | Casual alien |
| Malvaceae | *Alcea rosea* L. | H scap | Turkey | Temperate | Archaeophyte | Naturalized alien |
| Casuarinaceae | *Allocasuarina torulosa* (Aiton) L.A.S.Johnson | P scap | Australia | Tropical | | Cultivated |
| Casuarinaceae | *Allocasuarina verticillata* (Lam.) L.A.S.Johnson | P scap | Australia | Subtropical | Neophyte | Casual alien |
| Betulaceae | *Alnus cordata* Desf. | P scap | Corse | Temperate | | Native |
| Araceae | *Alocasia macrorrhizos* (L.) G.Don | G rhiz | S-E Asia, Queensland | Tropical | | Cultivated |
| Asphodelaceae | *Aloe ×caesia* Salm-Dyck | P succ | Cape Province | Subtropical | Neophyte | Naturalized alien |
| Asphodelaceae | *Aloe africana* Mill. | P succ | Cape Province | Subtropical | Neophyte | Casual alien |
| Asphodelaceae | *Aloe arborescens* Mill. | P succ | S Africa | Shrubland | Neophyte | Naturalized alien |
| Asphodelaceae | *Aloe arborescens* var. *frutescens* (Salm-Dyck) Link | P succ | S Africa | Shrubland | Neophyte | Naturalized alien |
| Asphodelaceae | *Aloe brachystachys* Baker | P succ | Tanzania | Tropical | Neophyte | Casual alien |
| Asphodelaceae | *Aloe distans* Haw. | P succ | Cape Province | Subtropical | | Cultivated |
| Asphodelaceae | *Aloe humilis* (L.) Mill. | P succ | Cape Province | Subtropical | Neophyte | Casual alien |
| Asphodelaceae | *Aloe marlothii* A.Berger | P succ | S Africa | Shrubland | | Cultivated |
| Asphodelaceae | *Aloe microstigma* Salm-Dyck | P succ | S Africa | Shrubland | | Cultivated |
| Asphodelaceae | *Aloe succotrina* Weston | P succ | Cape Province | Subtropical | | Cultivated |

**Table S1.** List of the Sicilian ornamental taxa per Families, Growth Form (according to [41, 42]), Geographical Origin (derived from [41]), Biome Origin (according to [41]), Resident Time and Status (Native/Alien) in Italy (derived both from [43,44]). New records from Sicily are reported with an asterisk.

| FAMILIES | TAXA | GROWTH FORM | GEOGRAPHICAL ORIGIN | BIOME ORIGIN | RESIDENT TIME | STATUS IN ITALY |
|---|---|---|---|---|---|---|
| Asphodelaceae | *Aloe vera* (L.) Burm.f. | P succ, NP | Oman | Shrubland | Archaeophyte | Naturalized alien |
| Asphodelaceae | *Aloiampelos ciliaris* (Haw.) Klopper & Gideon F.Sm. | Ch succ | Cape Province | Subtropical | Neophyte | Casual alien |
| Asphodelaceae | *Aloidendron barberae* (Dyer) Klopper & Gideon F.Sm. | P succ | S Africa | Tropical | | Cultivated |
| Verbenaceae | *Aloysia citrodora* Paláu | P caesp | S America | Subtropical | Neophyte | Casual alien |
| Verbenaceae | *Aloysia decipiens* Ravenna | P caesp | S America | Subtropical | | Cultivated |
| Zingiberaceae | *Alpinia zerumbet* (Pers.) B.L.Burtt & R.M.Sm. | G rhiz | S-E Asia | Subtropical | Neophyte | Invasive alien |
| Malvaceae | *Alyogyne huegelii* (Endl.) Fryxell* | P caesp | S-W Australia | Subtropical | | Cultivated |
| Amaranthaceae | *Amaranthus cruentus* L. | T scap | S America | Tropical | | Cultivated |
| Amaryllidaceae | *Amaryllis belladonna* L. | G bulb | S Africa | Subtropical | Neophyte | Naturalized alien |
| Rosaceae | *Amelanchier laevis* Wiegand | P scap | N America | Temperate | | Cultivated |
| Bignoniaceae | *Amphilophium buccinatorium* (DC.) L.G.Lohmann | P lian | C America | Tropical | | Cultivated |
| Annonaceae | *Annona cherimola* Mill.* | P scap | S-W America | Tropical | Neophyte | Casual alien |
| Basellaceae | *Anredera cordifolia* (Ten.) Steenis | G tub | S America | Tropical | Neophyte | Invasive alien |
| Fabaceae | *Anthyllis barba-jovis* L. | P caesp | W-C Mediterranean | Subtropical | | Native |
| Polygonaceae | *Antigonon leptopus* Hook. & Arn. | P lian | Mexico | Tropical | | Cultivated |
| Plantaginaceae | *Antirrhinum majus* L. | Ch frut | Spain, France | Temperate | Archaeophyte | Naturalized alien |
| Araucariaceae | *Araucaria araucana* (Molina) K.Koch | P scap | S America | Temperate | | Cultivated |
| Araucariaceae | *Araucaria bidwillii* Hook. | P scap | Australia | Subtropical | | Cultivated |
| Araucariaceae | *Araucaria columnaris* (G.Forst.) Hook. | P scap | New Caledonia | Tropical | | Cultivated |
| Araucariaceae | *Araucaria cunninghamii* Mudie | P scap | Australia | Subtropical | | Cultivated |
| Araucariaceae | *Araucaria heterophylla* (Salisb.) Franco | P scap | Norfolk Is. | Tropical | | Cultivated |
| Araucariaceae | *Araucaria luxurians* (Brongn. & Gris) de Laub. | P scap | C-S-E New Caledonia | Tropical | | Cultivated |
| Araucariaceae | *Araucaria rulei* F.Muell. | P scap | New Caledonia | Tropical | | Cultivated |
| Apocynaceae | *Araujia sericifera* Brot. | P lian | S America | Subtropical | Neophyte | Invasive alien |
| Ericaceae | *Arbutus unedo* L. | P scap | Mediterranean | Temperate | | Native |
| Arecaceae | *Archontophoenix alexandrae* (F.Muell.) H.Wendl. & Drude* | P scap | Queensland | Tropical | | Cultivated |
| Arecaceae | *Archontophoenix cunninghamiana* (H.Wendl.) H.Wendl. & Drude* | P scap | E Australia | Tropical | | Cultivated |
| Asteraceae | *Argyranthemum broussonetii* (Balb. ex Pers.) Humphries | Ch frut | Canary Is. | Subtropical | | Cultivated |
| Asteraceae | *Argyranthemum frutescens* (L.) Sch.Bip. | Ch frut | Canary Is. | Subtropical | Neophyte | Casual alien |
| Aristolochiaceae | *Aristolochia gigantea* Mart. & Zucc.* | P lian | S America | Subtropical | | Cultivated |
| Aristolochiaceae | *Aristolochia sempervirens* L. | P lian | Sicily, E Mediterranean | Tropical | | Native |
| Asteraceae | *Artemisia arborescens* L. | NP | C-S Mediterranean | Temperate | | Native |
| Poaceae | *Arundo donax* L. | G rhiz | S Asia | Subtropical | Archaeophyte | Invasive alien |

**Table S1.** List of the Sicilian ornamental taxa per Families, Growth Form (according to [41, 42]), Geographical Origin (derived from [41]), Biome Origin (according to [41]), Resident Time and Status (Native/Alien) in Italy (derived both from [43,44]). New records from Sicily are reported with an asterisk.

| FAMILIES | TAXA | GROWTH FORM | GEOGRAPHICAL ORIGIN | BIOME ORIGIN | RESIDENT TIME | STATUS IN ITALY |
|---|---|---|---|---|---|---|
| Apocynaceae | *Asclepias mellodora* A.St.-Hil.* | P scap | S America | Subtropical | | Cultivated |
| Asparagaceae | *Asparagus densiflorus* (Kunth) Jessop | G rhiz | S Africa | Subtropical | | Cultivated |
| Asparagaceae | *Asparagus falcatus* L. | P lian | S-E Africa, India | Tropical | Neophyte | Casual alien |
| Asparagaceae | *Asparagus setaceus* (Kunth) Jessop | G rhiz | S-E Africa | Tropical | Neophyte | Naturalized alien |
| Asparagaceae | *Aspidistra elatior* Blume | G rhiz | Japan | Subtropical | Neophyte | Casual alien |
| Aspleniaceae | *Asplenium antiquum* Makino | G rhiz | China, Korea | Subtropical | | Cultivated |
| Aspleniaceae | *Asplenium nitidum* Sw. | G rhiz | S-E Asia | Tropical | | Cultivated |
| Cactaceae | *Astrophytum myriostigma* Lem.* | P succ | Mexico | Shrubland | | Cultivated |
| Cactaceae | *Astrophytum ornatum* (DC.) Britton & Rose* | P succ | Mexico | Shrubland | | Cultivated |
| Amaranthaceae | *Atriplex halimus* L. | P caesp | Mediterranean, C-E Africa | Subtropical | | Native |
| Garryaceae | *Aucuba japonica* Thunb. | P caesp | E Asia | Temperate | Neophyte | Casual alien |
| Cactaceae | *Austrocylindropuntia cylindrica* (Lam.) Backeb. | P succ | S-W America | Subtropical | Neophyte | Naturalized alien |
| Cactaceae | *Austrocylindropuntia subulata* (Muehlenpf.) Backeb. | P succ | S America | Shrubland | Neophyte | Invasive alien |
| Poaceae | *Bambusa vulgaris* Schrad. ex J.C.Wendl. | P scap | China | Tropical | | Cultivated |
| Poaceae | *Bambusa vulgaris* Schrad. ex J.C.Wendl. 'Variegata' | P scap | Horticultural | Tropical | | Cultivated |
| Asteraceae | *Bartlettina sordida* (Less.) R.M.King & H.Rob. | P caesp, P scap | C America | Tropical | | Cultivated |
| Amaranthaceae | *Bassia scoparia* (L.) Beck | T scap | E Europe, Asia | Temperate | Neophyte | Invasive alien |
| Fabaceae | *Bauhinia forficata* Link | P scap | S America | Tropical | | Cultivated |
| Fabaceae | *Bauhinia purpurea* L. | P scap | S Asia | Tropical | Neophyte | Casual alien |
| Fabaceae | *Bauhinia variegata* L. | P scap | S Asia | Tropical | Neophyte | Casual alien |
| Asparagaceae | *Beaucarnea recurvata* (K.Koch & Fintelm.) Lem. | P scap | Mexico | Shrubland | | Cultivated |
| Asparagaceae | *Beaucarnea stricta* (K.Koch & Fintelm.) Lem. | P scap | Mexico | Tropical | | Cultivated |
| Begoniaceae | *Begonia cucullata* Willd. | H scap | S America | Tropical | | Cultivated |
| Berberidaceae | *Berberis fortunei* Lindl. | Ch suffr | China, Taiwan | Temperate | | Cultivated |
| Berberidaceae | *Berberis repens* Lindl. | Ch suffr | N America | Temperate | | Cultivated |
| Berberidaceae | *Berberis vulgaris* L. | NP | Europe, N-W Africa, W Asia | Temperate | | Native |
| Saxifragaceae | *Bergenia crassifolia* (L.) Fritsch | Ch caesp | C-N Asia | Temperate | Neophyte | Casual alien |
| Arecaceae | *Bismarckia nobilis* Hildebrandt & H.Wendl. | P scap | Madagascar | Tropical | | Cultivated |
| Amaranthaceae | *Bosea yervamora* L. | P caesp | Canary Is. | Subtropical | | Cultivated |
| Nyctaginaceae | *Bougainvillea glabra* Choisy | P lian | C-S America | Tropical | Neophyte | Casual alien |
| Nyctagynaceae | *Bougainvillea glabra* var. *sanderiana* Bosschere | P lian | C-S America | Tropical | Neophyte | Casual alien |
| Nyctagynaceae | *Bougainvillea sanderiana* (Dimmock) W. Falc. bis | P lian | C-S America | Tropical | | Cultivated |
| Nyctagynaceae | *Bougainvillea spectabilis* Willd. | P lian | C-S America | Tropical | Neophyte | Casual alien |

**Table S1.** List of the Sicilian ornamental taxa per Families, Growth Form (according to [41, 42]), Geographical Origin (derived from [41]), Biome Origin (according to [41]), Resident Time and Status (Native/Alien) in Italy (derived both from [43,44]). New records from Sicily are reported with an asterisk.

| FAMILIES | TAXA | GROWTH FORM | GEOGRAPHICAL ORIGIN | BIOME ORIGIN | RESIDENT TIME | STATUS IN ITALY |
|---|---|---|---|---|---|---|
| Malvaceae | *Brachychiton ×vinicolor* Guymer | P scap | Australia | Tropical | | Cultivated |
| Malvaceae | *Brachychiton acerifolius* (A.Cunn. ex G.Don) F.Muell. | P scap | E Australia | Tropical | | Cultivated |
| Malvaceae | *Brachychiton discolor* F.Muell. | P scap | Australia | Tropical | Neophyte | Casual alien |
| Malvaceae | *Brachychiton diversifolius* R.Br.* | P scap | Australia | Tropical | Neophyte | Casual alien |
| Malvaceae | *Brachychiton luridus* F.Muell. | P scap | Australia | Tropical | | Cultivated |
| Malvaceae | *Brachychiton populneus* (Schott & Endl.) R.Br. | P scap | E Australia | Subtropical | Neophyte | Casual alien |
| Malvaceae | *Brachychiton rupestris* (Lindl.) K.Schum. | P scap | Australia | Tropical | | Cultivated |
| Arecaceae | *Brahea armata* S.Watson | P scap | Mexico | Shrubland | Neophyte | Casual alien |
| Arecaceae | *Brahea calcarea* Liebm. | P scap | C America | Tropical | | Cultivated |
| Arecaceae | *Brahea dulcis* (Kunth) Mart. | P scap | C America | Tropical | | Cultivated |
| Moraceae | *Broussonetia papyrifera* (L.) Vent. | P scap | E Asia | Temperate | Neophyte | Invasive alien |
| Solanaceae | *Brugmansia arborea* (L.) Sweet | P scap | C-S America | Tropical | | Cultivated |
| Solanaceae | *Brugmansia aurea* Lagerh. | P caesp, P scap | S America | Tropical | Neophyte | Casual alien |
| Solanaceae | *Brugmansia suaveolens* (Humb. & Bonpl. ex Willd.) Sweet | P caesp, P scap | Brazil | Tropical | Neophyte | Casual alien |
| Scrophulariaceae | *Buddleja davidii* Franch. | P caesp | China | Temperate | Neophyte | Invasive alien |
| Loganiaceae | *Buddleja madagascariensis* Lam. | P caesp | Madagascar | Subtropical | Neophyte | Casual alien |
| Arecaceae | *Butia capitata* (Mart.) Becc. | P scap | Brazil | Tropical | | Cultivated |
| Arecaceae | *Butia yatay* (Mart.) Becc. | P scap | Brazil, N Argentina | Subtropical | | Cultivated |
| Buxaceae | *Buxus balearica* Lam. | P caesp | W Mediterranean, Turkey | Subtropical | | Native |
| Buxaceae | *Buxus microphylla* Siebold & Zucc. | P caesp | Japan | Temperate | Neophyte | Casual alien |
| Buxaceae | *Buxus rotundifolia* (Britton) Mathou | P caesp | Cuba | Tropical | | Cultivated |
| Buxaceae | *Buxus sempervirens* L. | NP | C-S Europe, N Africa, Caucasus | Temperate | | Native |
| Asteraceae | *Calendula officinalis* L. | T scap | S Europa | Temperate | Archaeophyte | Naturalized alien |
| Asteraceae | *Calendula suffruticosa* Vahl | Ch suffr | S-W Mediterranean | Subtropical | | Native |
| Fabaceae | *Calliandra tweediei* Benth. | P caesp, P scap | S America | Tropical | | Cultivated |
| Cupressaceae | *Calocedrus decurrens* (Torr.) Florin | P scap | Oregon, California, Mexico | Temperate | Neophyte | Naturalized alien |
| Calycanthaceae | *Calycanthus floridus* L. | P caesp | N America | Temperate | Neophyte | Casual alien |
| Calycanthaceae | *Calycanthus floridus* var. *glaucus* (Willd.) Torr. & A.Gray | P caesp | N America | Temperate | Neophyte | Casual alien |
| Convolvulaceae | *Calystegia sepium* (L.) R.Br. | H scand | Temperate, Subtropics | Temperate | | Native |
| Theaceae | *Camellia japonica* L. | P scap | S-E Asia | Subtropical | | Cultivated |
| Theaceae | *Camellia sinensis* (L.) Kuntze | P scap | S Asia | Subtropical | | Cultivated |
| Lauraceae | *Camphora officinarum* Boerh. ex Fabr. | P scap | Korea, Japan | Subtropical | | Cultivated |
| Bignoniaceae | *Campsis grandiflora* (Thunb.) K.Schum. | P lian | China, Japan | Temperate | | Cultivated |

**Table S1.** List of the Sicilian ornamental taxa per Families, Growth Form (according to [41, 42]), Geographical Origin (derived from [41]), Biome Origin (according to [41]), Resident Time and Status (Native/Alien) in Italy (derived both from [43,44]). New records from Sicily are reported with an asterisk.

| FAMILIES | TAXA | GROWTH FORM | GEOGRAPHICAL ORIGIN | BIOME ORIGIN | RESIDENT TIME | STATUS IN ITALY |
|---|---|---|---|---|---|---|
| Bignoniaceae | *Campsis grandiflora* (Thunb.) K.Schum. 'Flava' | P lian | China, Japan | Temperate | | Cultivated |
| Bignoniaceae | *Campsis radicans* (L.) Bureau | P lian | S-E USA | Subtropical | Neophyte | Naturalized alien |
| Cannaceae | *Canna indica* L. | G rhiz | C-S America | Tropical | Neophyte | Naturalized alien |
| Capparaceae | *Capparis spinosa* L. | NP | Mediterranean | Subtropical | | Native |
| Solanaceae | *Capsicum annuum* L. | T scap | S America | Tropical | Neophyte | Casual alien |
| Sapindaceae | *Cardiospermum grandiflorum* Sw. | P lian | C-S America, C-S Africa | Tropical | Neophyte | Naturalized alien |
| Sapindaceae | *Cardiospermum halicacabum* L. | P lian | Tropics, Subtropics | Tropical | Neophyte | Naturalized alien |
| Caricaceae | *Carica papaya* L. | P scap | C-S America | Tropical | | Cultivated |
| Apocynaceae | *Carissa bispinosa* (L.) Desf. ex Brenan | P caesp | S Africa | Tropical | | Cultivated |
| Apocynaceae | *Carissa macrocarpa* (Eckl.) A.DC. | P caesp | S Africa | Shrubland | Neophyte | Naturalized alien |
| Aizoaceae | *Carpobrotus acinaciformis* (L.) L.Bolus | Ch suffr | S Africa | Subtropical | Neophyte | Invasive alien |
| Aizoaceae | *Carpobrotus edulis* (L.) N.E.Br. | Ch suffr | S Africa | Subtropical | Neophyte | Invasive alien |
| Juglandaceae | *Carya illinoinensis* (Wangenh.) K.Koch | P scap | C-E USA, Mexico | Temperate | Neophyte | Casual alien |
| Apocynaceae | *Cascabela thevetia* (L.) Lippold | P caesp, P scap | C-S America | Tropical | Neophyte | Naturalized alien |
| Rutaceae | *Casimiroa edulis* La Llave | P scap | C America | Tropical | | Cultivated |
| Fagaceae | *Castanea sativa* Mill. | P scap | Balkan, Caucasus | Temperate | | Native |
| Casuarinaceae | *Casuarina equisetifolia* L. | P scap | E Asia, Australia | Tropical | Neophyte | Naturalized alien |
| Casuarinaceae | *Casuarina glauca* Sieber ex Spreng. | P scap | Australia | Subtropical | | Cultivated |
| Bignoniaceae | *Catalpa bignonioides* Walter | P scap | N America | Temperate | Neophyte | Naturalized alien |
| Apocynaceae | *Catharanthus roseus* (L.) G.Don | Ch frut | Madagascar | Tropical | Neophyte | Naturalized alien |
| Pinaceae | *Cedrus atlantica* (Endl.) Manetti ex Carrière | P scap | N-W Africa | Temperate | Neophyte | Naturalized alien |
| Pinaceae | *Cedrus deodara* (Roxb. ex D.Don) G.Don | P scap | C Asia | Temperate | Neophyte | Naturalized alien |
| Pinaceae | *Cedrus libani* A.Rich. | P scap | Turkey, Lebanon | Temperate | Neophyte | Casual alien |
| Malvaceae | *Ceiba insignis* (Kunth) P.E.Gibbs & Semir | P scap | S America | Tropical | | Cultivated |
| Malvaceae | *Ceiba speciosa* (A.St.-Hil., A.Juss. & Cambess.) Ravenna | P scap | S America | Tropical | Neophyte | Casual alien |
| Cannabaceae | *Celtis australis* L. | P scap | S Europe, N-W Africa, Caucasus | Subtropical | | Native |
| Cannabaceae | *Celtis bungeana* Blume | P scap | China, Korea | Temperate | | Cultivated |
| Poaceae | *Cenchrus setaceus* (Forssk.) Morrone | H caesp | Africa, S-W Asia | Tropical | Neophyte | Invasive alien |
| Cephalotaxaceae | *Cephalotaxus fortunei* Hook. | P caesp | E Asia | Temperate | | Cultivated |
| Cephalotaxaceae | *Cephalotaxus harringtonia* (Knight ex J.Forbes) K.Koch | P caesp, P scap | E Asia | Temperate | Neophyte | Casual alien |
| Fabaceae | *Ceratonia siliqua* L. | P scap | Mediterranean, Caucasus | Subtropical | | Native |
| Zamiaceae | *Ceratozamia mexicana* Brongn. | P caesp, P scap | Mexico | Tropical | | Cultivated |
| Fabaceae | *Cercis siliquastrum* L. | P scap | S-E Europe | Temperate | | Native |

**Table S1.** List of the Sicilian ornamental taxa per Families, Growth Form (according to [41, 42]), Geographical Origin (derived from [41]), Biome Origin (according to [41]), Resident Time and Status (Native/Alien) in Italy (derived both from [43,44]). New records from Sicily are reported with an asterisk.

| FAMILIES | TAXA | GROWTH FORM | GEOGRAPHICAL ORIGIN | BIOME ORIGIN | RESIDENT TIME | STATUS IN ITALY |
|---|---|---|---|---|---|---|
| Cactaceae | *Cereus hildmannianus* K.Schum. | P succ | S America | Tropical | Neophyte | Naturalized alien |
| Cactaceae | *Cereus jamacaru* DC. | P succ | Brazil | Tropical | | Cultivated |
| Cactaceae | *Cereus repandus* (L.) Mill. | P succ | C-S America | Tropical | | Cultivated |
| Cactaceae | *Cereus repandus* (L.) Mill. 'Monstruosus'* | P succ | Horticultural | Tropical | | Cultivated |
| Cactaceae | *Cereus stenogonus* K.Schum. | P succ | S America | Tropical | | Cultivated |
| Cactaceae | *Cereus validus* Haw. | P succ | S America | Tropical | | Cultivated |
| Solanaceae | *Cestrum aurantiacum* Lindl. | P caesp | C-S America | Tropical | | Cultivated |
| Solanaceae | *Cestrum elegans* (Brongn. ex Neumann) Schltdl. | P caesp | Mexico | Tropical | | Cultivated |
| Solanaceae | *Cestrum nocturnum* L. | P caesp | C-S America | Tropical | | Cultivated |
| Solanaceae | *Cestrum parqui* (Lam.) L'Hér. | P caesp | S America | Subtropical | Neophyte | Invasive alien |
| Solanaceae | *Cestrum thyrsoideum* Kunth | P caesp | Mexico | Tropical | | Cultivated |
| Rosaceae | *Chaenomeles speciosa* (Sweet) Nakai | P scap | China | Temperate | Neophyte | Casual alien |
| Cactaceae | *Chamaecereus silvestrii* (Speg.) Britton & Rose | Ch succ | Argentina | Shrubland | Neophyte | Casual alien |
| Cupressaceae | *Chamaecyparis lawsoniana* (A.Murray bis) Parl. | P scap | N America | Temperate | Neophyte | Casual alien |
| Arecaceae | *Chamaedorea elatior* Mart. | P caesp | C America | Tropical | Neophyte | Casual alien |
| Arecaceae | *Chamaedorea elegans* Mart. | P caesp | C America | Tropical | | Cultivated |
| Arecaceae | *Chamaedorea oblongata* Mart.* | P caesp | C America | Tropical | | Cultivated |
| Arecaceae | *Chamaerops humilis* L. | NP | Mediterranean | Subtropical | | Native |
| Iridaceae | *Chasmanthe aethiopica* (L.) N.E.Br. | G bulb | Cape Province | Subtropical | Neophyte | Invasive alien |
| Asteraceae | *Cheirolophus crassifolius* (Bertol.) Susanna | H scap | Malta | Subtropical | | Cultivated |
| Calycanthaceae | *Chimonanthus praecox* (L.) Link | P caesp | China | Temperate | Neophyte | Casual alien |
| Asparagaceae | *Chlorophytum comosum* (Thunb.) Jacques | H rept | C-S Africa | Tropical | Neophyte | Naturalized alien |
| Asparagaceae | *Chlorophytum comosum* (Thunb.) Jacques 'Vittatum' | H caesp | Horticultural | Tropical | | Cultivated |
| Asteraceae | *Chrysanthemum indicum* L. | Ch frut | E Asia | Temperate | | Cultivated |
| Oleaceae | *Chrysojasminum floridum* (Bunge) Banfi | P caesp | China | Temperate | | Cultivated |
| Oleaceae | *Chrysojasminum fruticans* (L.) Banfi | P caesp | Mediterranean, Caucasus | Temperate | | Native |
| Vitaceae | *Cissus erosa* Rich. | P lian | C-S America | Tropical | | Cultivated |
| Rutaceae | *Citrus ×aurantium* f. *aurantium* | P scap | China | Subtropical | Archaeophyte | Casual alien |
| Rutaceae | *Citrus ×aurantium* L. | P scap | S-E Asia | Subtropical | Archaeophyte | Casual alien |
| Rutaceae | *Citrus ×limon* (L.) Osbeck | P scap | Artificial hybrid | Subtropical | Archaeophyte | Casual alien |
| Rutaceae | *Citrus ×limon* (L.) Osbeck 'Lunario' | P scap | Artificial hybrid | Subtropical | | Cultivated |
| Rutaceae | *Citrus ×lumia* Risso | P scap | Artificial hybrid | Subtropical | Archaeophyte | Casual alien |
| Rutaceae | *Citrus japonica* Thunb. | P scap | China | Subtropical | | Cultivated |

**Table S1.** List of the Sicilian ornamental taxa per Families, Growth Form (according to [41, 42]), Geographical Origin (derived from [41]), Biome Origin (according to [41]), Resident Time and Status (Native/Alien) in Italy (derived both from [43,44]). New records from Sicily are reported with an asterisk.

| FAMILIES | TAXA | GROWTH FORM | GEOGRAPHICAL ORIGIN | BIOME ORIGIN | RESIDENT TIME | STATUS IN ITALY |
|---|---|---|---|---|---|---|
| Rutaceae | *Citrus maxima* (Burm.) Merr.* | P scap | Indo-China | Tropical | | Cultivated |
| Rutaceae | *Citrus reticulata* Blanco | P scap | China | Subtropical | | Cultivated |
| Rutaceae | *Citrus trifoliata* L. | P scap | China | Temperate | Neophyte | Casual alien |
| Cactaceae | *Cleistocactus strausii* (Heese) Backeb. | P succ | Bolivia | Shrubland | Neophyte | Casual alien |
| Ranunculaceae | *Clematis ×jackmanii* T.Moore | P lian | Artificial hybrid | Temperate | | Cultivated |
| Ranunculaceae | *Clematis cirrhosa* L. | P lian | Mediterranean | Temperate | | Native |
| Ranunculaceae | *Clematis recta* L. | P lian | S Europa | Temperate | | Native |
| Lamiaceae | *Clerodendrum chinense* (Osbeck) Mabb. | P caesp | C-S-E Asia | Tropical | | Cultivated |
| Amaryllidaceae | *Clivia miniata* Regel | G rhiz | S Africa | Subtropical | | Cultivated |
| Fabaceae | *Cochliasanthus caracalla* (L.) Trew* | P lian | C-S America | Tropical | | Cultivated |
| Euphorbiaceae | *Codiaeum variegatum* (L.) Rumph. ex A.Juss. | P caesp | S-E Asia, E Australia | Tropical | | Cultivated |
| Lamiaceae | *Coleus scutellarioides* (L.) Benth. | H scap | S-E Asia, Australia | Tropical | Neophyte | Casual alien |
| Araceae | *Colocasia esculenta* (L.) Schott. | G rhiz | Sri Lanka, India, Malaysia | Tropical | Archaeophyte | Naturalized alien |
| Combretaceae | *Combretum indicum* (L.) De Filipps | P lian | Tanzania, Tropical Asia, Australia | Tropical | | Cultivated |
| Cactaceae | *Consolea rubescens* (Salm-Dyck ex DC.) Lem. | P succ | C America | Tropical | | Cultivated |
| Convolvulaceae | *Convolvulus scammonia* L. | H scand | Caucasus | Subtropical | | Cultivated |
| Boraginaceae | *Cordia francisci* Ten. | P scap | Unknown | Tropical | | Cultivated |
| Boraginaceae | *Cordia myxa* L. | P scap | C-S Asia | Tropical | | Cultivated |
| Asparagaceae | *Cordyline australis* (G.Forst.) Endl. | P caesp | New Zealand | Subtropical | Neophyte | Casual alien |
| Rosaceae | *Cormus domestica* (L.) Spach | P scap | C-S Europe, N Africa, Caucasus | Temperate | | Native |
| Poaceae | *Cortaderia selloana* (Schult. & Schult.f.) Asch. & Graebn. | H caesp | S America | Subtropical | Neophyte | Invasive alien |
| Betulaceae | *Corylus avellana* L. | P caesp | Europe, Caucasus | Temperate | | Native |
| Myrtaceae | *Corymbia citriodora* (Hook.) K.D.Hill & … | P scap | Queensland | Tropical | | Cultivated |
| Corynocarpaceae | *Corynocarpus laevigatus* J.R.Forst. & G.Forst. | P caesp, P scap | New Zealand | Subtropical | | Cultivated |
| Anacardiaceae | *Cotinus coggygria* Scop. | P caesp, P scap | C-S Europe, C Asia | Temperate | | Native |
| Rosaceae | *Cotoneaster pannosus* Franch. | P caesp | China | Temperate | Neophyte | Naturalized alien |
| Rosaceae | *Cotoneaster simonsii* Baker | P caesp | E Asia | Temperate | Neophyte | Naturalized alien |
| Crassulaceae | *Cotyledon orbiculata* L. | NP succ | S Africa | Shrubland | Neophyte | Casual alien |
| Crassulaceae | *Cotyledon orbiculata* var. *oblonga* (Haw.) DC. | NP succ | S Africa | Shrubland | Neophyte | Casual alien |
| Crassulaceae | *Crassula arborescens* (Mill.) Willd. | NP | Cape Province | Subtropical | | Cultivated |
| Crassulaceae | *Crassula muscosa* L. | NP succ | S Africa | Subtropical | Neophyte | Invasive alien |
| Crassulaceae | *Crassula ovata* (Mill.) Druce | NP succ | S Africa | Subtropical | Neophyte | Casual alien |
| Crassulaceae | *Crassula perfoliata* var. *falcata* (J.C.Wendl.) Toelken | NP succ | Cape Province | Subtropical | | Cultivated |

**Table S1.** List of the Sicilian ornamental taxa per Families, Growth Form (according to [41, 42]), Geographical Origin (derived from [41]), Biome Origin (according to [41]), Resident Time and Status (Native/Alien) in Italy (derived both from [43,44]). New records from Sicily are reported with an asterisk.

| FAMILIES | TAXA | GROWTH FORM | GEOGRAPHICAL ORIGIN | BIOME ORIGIN | RESIDENT TIME | STATUS IN ITALY |
|---|---|---|---|---|---|---|
| Crassulaceae | *Crassula rupestris* L.f. | NP succ | S Africa | Subtropical | | Cultivated |
| Crassulaceae | *Crassula tetragona* L. | NP succ | Cape Province | Subtropical | | Cultivated |
| Rosaceae | *Crataegus azarolus* L. | P caesp, P scap | Mediterranean, Caucasus | Subtropical | Archaeophyte | Naturalized alien |
| Rosaceae | *Crataegus monogyna* Jacq. | P caesp | Europe, N-W Africa, Caucasus | Temperate | | Native |
| Amaryllidaceae | *Crinum ×powellii* Baker | G bulb | Artificial hybrid | Tropical | | Cultivated |
| Amaryllidaceae | *Crinum latifolium* L. | G bulb | India, China | Tropical | | Cultivated |
| Cupressaceae | *Cryptomeria japonica* (Thunb. ex L.f.) D.Don | P scap | Japan | Temperate | Neophyte | Naturalized alien |
| Lythraceae | *Cuphea hyssopifolia* Kunth | Ch suffr | C America | Tropical | Neophyte | Casual alien |
| Lythraceae | *Cuphea melvilla* Lindi. | Ch suffr | S America | Tropical | | Cultivated |
| Cupressaceae | *Cupressus sempervirens* f. *horizontalis* (Mill.) Voss | P scap | Mediterranean, Iran | Temperate | Archaeophyte | Naturalized alien |
| Cupressaceae | *Cupressus sempervirens* L. | P scap | Mediterranean, Iran | Temperate | Archaeophyte | Naturalized alien |
| Asteraceae | *Curio ficoides* (L.) P.V.Heath | Ch suffr | Cape Province | Subtropical | | Cultivated |
| Asteraceae | *Curio rowleyanus* (H.Jacobsen) P.V.Heath* | Ch suffr | Cape Province | Subtropical | | Cultivated |
| Asteraceae | *Curio talinoides* var. *mandraliscae* (Tineo) P.V.Heath | Ch suffr | Cape Province | Shrubland | Neophyte | Casual alien |
| Cycadaceae | *Cycas circinalis* L. | P scap | S India | Tropical | | Cultivated |
| Cycadaceae | *Cycas revoluta* Thunb. | P caesp | China, Japan, Taiwan | Subtropical | Neophyte | Casual alien |
| Primulaceae | *Cyclamen persicum* Mill. | G bulb | Algeria, E Mediterranean | Subtropical | Neophyte | Naturalized alien |
| Rosaceae | *Cydonia oblonga* Mill. | P scap | S-W Asia | Temperate | Archaeophyte | Naturalized alien |
| Cactaceae | *Cylindropuntia imbricata* (Haw.) F.M.Knuth | P succ | USA, C America | Shrubland | Neophyte | Naturalized alien |
| Cyperaceae | *Cyperus alternifolius* L. | G rhiz | Madagascar | Tropical | Neophyte | Invasive alien |
| Cyperaceae | *Cyperus papyrus* L. | G rhiz | Africa, Israel | Tropical | Archaeophyte | Naturalized alien |
| Polypodiaceae | *Cyrtomium falcatum* (L.f.) C.Presl | G rhiz | China, S-E Asia | Temperate | Neophyte | Naturalized alien |
| Fabaceae | *Cytisus scoparius* (L.) Link | P caesp | Europe | Temperate | | Native |
| Fabaceae | *Cytisus villosus* Pourr. | P caesp | Mediterranean | Subtropical | | Native |
| Asteraceae | *Dahlia ×hortensis* Guillaumin | G rhiz | Horticultural | Tropical | | Cultivated |
| Asteraceae | *Dahlia pinnata* Cav. | G rhiz | Mexico | Tropical | | Cultivated |
| Asparagaceae | *Dasylirion glaucophyllum* Hook. | P caesp | Mexico | Shrubland | | Cultivated |
| Solanaceae | *Datura sanguinea* Ruiz & Pav. | P scap | S America | Tropical | | Cultivated |
| Solanaceae | *Datura versicolor* (Lagerh.) Saff. | P caesp, P scap | C America | Tropical | | Cultivated |
| Solanaceae | *Datura wrightii* Regel | T scap | N America | Subtropical | Neophyte | Invasive alien |
| Lardizabalaceae | *Decaisnea fargesii* Franch. | P caesp, P scap | Nepal, China | Temperate | | Cultivated |
| Asteraceae | *Delairea odorata* Lem. | H rept | Cape Province | Tropical | Neophyte | Naturalized alien |
| Fabaceae | *Delonix regia* (Bojer) Raf. | P scap | Madagascar | Tropical | | Cultivated |

**Table S1.** List of the Sicilian ornamental taxa per Families, Growth Form (according to [41, 42]), Geographical Origin (derived from [41]), Biome Origin (according to [41]), Resident Time and Status (Native/Alien) in Italy (derived both from [43,44]). New records from Sicily are reported with an asterisk.

| FAMILIES | TAXA | GROWTH FORM | GEOGRAPHICAL ORIGIN | BIOME ORIGIN | RESIDENT TIME | STATUS IN ITALY |
|---|---|---|---|---|---|---|
| Ranunculaceae | *Delphinium consolida* L. | T scap | Europe, N-C Asia | Temperate | | Native |
| Fabaceae | *Dermatophyllum secundiflorum* (Ortega) Gandhi & Reveal | P caesp, P scap | C America | Shrubland | | Cultivated |
| Bromeliaceae | *Deuterocohnia brevifolia* (Griseb.) M.A.Spencer & L.B.Sm.* | P caesp | S America | Subtropical | | Cultivated |
| Hydrangeaceae | *Deutzia scabra* Thunb. | P caesp | Japan | Temperate | | Cultivated |
| Caryophyllaceae | *Dianthus caryophyllus* L. | H scap | Balkan | Temperate | | Native |
| Convolvulaceae | *Dichondra micrantha* Urb. | G rhiz | Cuba | Subtropical | Neophyte | Naturalized alien |
| Araceae | *Dieffenbachia seguine* (Jacq.) Schott | P caesp | S America | Tropical | | Cultivated |
| Plantaginaceae | *Digitalis purpurea* L. | H scap | S-W Europe, Morocco | Temperate | | Native |
| Asteraceae | *Dimorphotheca fruticosa* (L.) DC. | H scap | Cape Province | Subtropical | Neophyte | Casual alien |
| Asteraceae | *Dimorphotheca sinuata* DC. | H caesp | S Africa | Subtropical | | Cultivated |
| Zamiaceae | *Dioon edule* Lindl. | P scap | Mexico | Tropical | | Cultivated |
| Dioscoreaceae | *Dioscorea communis* (L.) Caddick & Wilkin | G rad | C-S Europe, N-W Africa, Caucasus | Temperate | | Native |
| Ebenaceae | *Diospyros kaki* Thunb. | P scap | E Asia | Temperate | Neophyte | Naturalized alien |
| Ebenaceae | *Diospyros lotus* L. | P scap | C Asia | Temperate | Neophyte | Naturalized alien |
| Bignoniaceae | *Dolichandra unguis-cati* (L.) L.G.Lohmann | P lian | C-S America | Tropical | Neophyte | Naturalized alien |
| Salicaceae | *Dovyalis afra* (Hook.f. & Harv.) Warb. | P scap | S Africa | Subtropical | | Cultivated |
| Asparagaceae | *Dracaena draco* L. | P scap | Canary Is. | Subtropical | Neophyte | Naturalized alien |
| Asparagaceae | *Dracaena trifasciata* (Prain) Mabb. | G rhiz | C Africa | Tropical | | Cultivated |
| Asparagaceae | *Dracaena trifasciata* (Prain) Mabb. 'Hahnii'* | G rhiz | Horticultural | Tropical | | Cultivated |
| Araceae | *Dracunculus vulgaris* Schott | G rhiz | S-E Europe, N-W Africa | Subtropical | | Native |
| Asparagaceae | *Drimia pancration* (Steinh.) J.C.Manning & Goldblatt | G bulb | Mediterranean | Subtropical | | Native |
| Aizoaceae | *Drosanthemum floribundum* (Haw.) Schwantes | Ch frut | S Africa | Shrubland | Neophyte | Naturalized alien |
| Aizoaceae | *Drosanthemum hispidum* (L.) Schwantes | Ch frut | Africa | Shrubland | Neophyte | Casual alien |
| Polypodiaceae | *Dryopteris pallida* (Bory) Maire & Petitm. | G rhiz | Mediterranean, Iran | Subtropical | | Native |
| Polypodiaceae | *Dryopteris villarii* (Bellardi) Woyn. ex Schinz & Thell. | G rhiz | C-S-E Europe, Caucasus | Temperate | | Native |
| Verbenaceae | *Duranta erecta* L. | P caesp | C-S America | Tropical | | Cultivated |
| Verbenaceae | *Duranta repens* f. variegata (L.H.Bailey) Moldenke | P caesp | C-S America | Tropical | | Cultivated |
| Bromeliaceae | *Dyckia brevifolia* Baker | Ch frut | Brazil | Subtropical | | Cultivated |
| Cucurbitaceae | *Ecballium elaterium* (L.) A.Rich. | T scap | Mediterranean, W Asia | Subtropical | | Native |
| Cactaceae | *Echinopsis oxygona* (Link) Zucc. ex Pfeiff. & Otto | P succ | S America | Subtropical | Neophyte | Casual alien |
| Boraginaceae | *Echium candicans* L.f. | P caesp | Madeira | Subtropical | Neophyte | Naturalized alien |
| Boraginaceae | *Ehretia tinifolia* L. | P scap | C America | Tropical | | Cultivated |
| Elaeagnaceae | *Elaeagnus ×submacrophylla* Servett. | P scap | Korea, Japan | Temperate | Neophyte | Casual alien |

**Table S1.** List of the Sicilian ornamental taxa per Families, Growth Form (according to [41, 42]), Geographical Origin (derived from [41]), Biome Origin (according to [41]), Resident Time and Status (Native/Alien) in Italy (derived both from [43,44]). New records from Sicily are reported with an asterisk.

| FAMILIES | TAXA | GROWTH FORM | GEOGRAPHICAL ORIGIN | BIOME ORIGIN | RESIDENT TIME | STATUS IN ITALY |
|---|---|---|---|---|---|---|
| Elaeagnaceae | *Elaeagnus angustifolia* L. | P scap | C Asia | Temperate | Neophyte | Naturalized alien |
| Elaeagnaceae | *Elaeagnus macrophylla* Thunb. | P scap | China, Japan | Subtropical | Neophyte | Casual alien |
| Elaeagnaceae | *Elaeagnus pungens* Thunb. | P scap | China | Temperate | Neophyte | Invasive alien |
| Celastraceae | *Elaeodendron australe* Vent. | P caesp, P scap | E Australia | Subtropical | | Cultivated |
| Proteaceae | *Embothrium coccineum* J.R.Forst. & G.Forst. | P caesp, P scap | S America | Temperate | | Cultivated |
| Fabaceae | *Enterolobium contortisiliquum* (Vell.) Morong | P scap | S America | Tropical | | Cultivated |
| Araceae | *Epipremnum aureum* (Linden & André) G.S.Bunting | P lian | Moorea | Tropical | | Cultivated |
| Scrophulariaceae | *Eremophila nivea* Chinnock* | P caesp | Australia | Subtropical | | Cultivated |
| Ericaceae | *Erica multiflora* L. | P caesp | Mediterranean | Temperate | | Native |
| Rosaceae | *Eriobotrya japonica* (Thunb.) Lindl. | P scap | China | Temperate | Neophyte | Naturalized alien |
| Asteraceae | *Eriocephalus africanus* L. | P caesp | Cape Province | Subtropical | Neophyte | Casual alien |
| Fabaceae | *Erythrina afra* Thunb. | P scap | S Africa | Subtropical | | Cultivated |
| Fabaceae | *Erythrina crista-galli* L. | P scap | S America | Subtropical | | Cultivated |
| Fabaceae | *Erythrina humeana* Spreng. | P scap | S Africa | Tropical | | Cultivated |
| Fabaceae | *Erythrostemon gilliesii* (Hook.) Klotzsch | P scap | S America | Temperate | Neophyte | Naturalized alien |
| Papaveraceae | *Eschscholzia californica* Cham.* | H scap, T scap | S-W USA, N Mexico | Temperate | Neophyte | Casual alien |
| Myrtaceae | *Eucalyptus bicolor* A.Cunn. | P scap | Australia | Shrubland | | Cultivated |
| Myrtaceae | *Eucalyptus bicolor* var. *parviflora* F.Muell. | P scap | Australia | Shrubland | | Cultivated |
| Myrtaceae | *Eucalyptus camaldulensis* Dehnh. | P scap | Australia | Shrubland | Neophyte | Invasive alien |
| Myrtaceae | *Eucalyptus globulus* Labill. | P scap | Australia | Temperate | Neophyte | Naturalized alien |
| Myrtaceae | *Eucalyptus grandis* W.Hill ex Maiden | P scap | Australia | Subtropical | | Cultivated |
| Myrtaceae | *Eucalyptus gunnii* Hook.f. | P scap | Australia | Temperate | | Cultivated |
| Myrtaceae | *Eucalyptus gunnii* Hook.f. 'Azura' | P scap | Horticultural | Temperate | | Cultivated |
| Myrtaceae | *Eucalyptus macrocarpa* Hook. | P scap | Australia | Subtropical | | Cultivated |
| Myrtaceae | *Eucalyptus robusta* Sm. | P scap | Australia | Subtropical | Neophyte | Casual alien |
| Myrtaceae | *Eucalyptus sideroxylon* A.Cunn. ex Woolls* | P scap | Australia | Subtropical | Neophyte | Casual alien |
| Myrtaceae | *Eugenia cerasiflora* Miq. | P scap | Brazil | Tropical | | Cultivated |
| Celastraceae | *Euonymus europaeus* L. | P scap | Europe, W Asia | Temperate | | Native |
| Celastraceae | *Euonymus fortunei* (Turcz.) Hand.-Mazz. | P caesp | C-E Asia | Temperate | Neophyte | Naturalized alien |
| Celastraceae | *Euonymus japonicus* Thunb. | P caesp | Japan | Subtropical | Neophyte | Naturalized alien |
| Celastraceae | *Euonymus japonicus* Thunb. 'Aureo variegatus' | P caesp | Japan | Subtropical | | Cultivated |
| Celastraceae | *Euonymus japonicus* Thunb. 'Medio-pictus' | P caesp | Japan | Subtropical | | Cultivated |
| Euphorbiaceae | *Euphorbia abyssinica* J.F.Gmel. | P succ | C Africa | Shrubland | | Cultivated |

**Table S1.** List of the Sicilian ornamental taxa per Families, Growth Form (according to [41, 42]), Geographical Origin (derived from [41]), Biome Origin (according to [41]), Resident Time and Status (Native/Alien) in Italy (derived both from [43,44]). New records from Sicily are reported with an asterisk.

| FAMILIES | TAXA | GROWTH FORM | GEOGRAPHICAL ORIGIN | BIOME ORIGIN | RESIDENT TIME | STATUS IN ITALY |
|---|---|---|---|---|---|---|
| Euphorbiaceae | *Euphorbia ammak* Schweinf. | P succ | Arabian Peninsula | Shrubland | | Cultivated |
| Euphorbiaceae | *Euphorbia canariensis* L. | P succ | Canary Is. | Subtropical | | Cultivated |
| Euphorbiaceae | *Euphorbia candelabrum* Welw. | P succ | Angola | Shrubland | | Cultivated |
| Euphorbiaceae | *Euphorbia dendroides* L. | NP | Mediterranean | Temperate | | Native |
| Euphorbiaceae | *Euphorbia grandicornis* Blanc* | P succ | S Africa | Shrubland | | Cultivated |
| Euphorbiaceae | *Euphorbia grandidens* Haw. | P succ | S Africa | Shrubland | | Cultivated |
| Euphorbiaceae | *Euphorbia ingens* E.Mey. ex Boiss. | P succ | C-S Africa | Tropical | | Cultivated |
| Euphorbiaceae | *Euphorbia lactea* Haw. | P succ | Sri Lanka | Tropical | | Cultivated |
| Euphorbiaceae | *Euphorbia marginata* Pursh | T scap | N America | Temperate | Neophyte | Casual alien |
| Euphorbiaceae | *Euphorbia milii* Des Moul. | P succ | Madagascar | Shrubland | | Cultivated |
| Euphorbiaceae | *Euphorbia pulcherrima* Willd. | NP | C America | Tropical | Neophyte | Casual alien |
| Euphorbiaceae | *Euphorbia ramipressa* Croizat | P succ | Madagascar | Shrubland | | Cultivated |
| Euphorbiaceae | *Euphorbia resinifera* Berg | P succ | Morocco | Subtropical | | Cultivated |
| Euphorbiaceae | *Euphorbia splendens* Bojer ex Hook. | P succ | Madagascar | Tropical | | Cultivated |
| Euphorbiaceae | *Euphorbia tirucalli* L. | P succ | Madagascar | Shrubland | | Cultivated |
| Euphorbiaceae | *Euphorbia triangularis* Desf. ex A.Berger | P succ | S Africa | Shrubland | | Cultivated |
| Euphorbiaceae | *Euphorbia umbellata* (Pax) Bruyns | P caesp, P scap | C Africa | Tropical | | Cultivated |
| Euphorbiaceae | *Euphorbia virosa* Willd. | P succ | S Africa | Shrubland | | Cultivated |
| Asteraceae | *Euryops pectinatus* (L.) Cass.* | P caesp | S Africa | Subtropical | | Cultivated |
| Polygonaceae | *Fallopia baldschuanica* (Regel) Holub | P lian | C Asia | Temperate | Neophyte | Invasive alien |
| Asteraceae | *Farfugium japonicum* (L.) Kitam. | H caesp | China, Japan | Subtropical | | Cultivated |
| Araliaceae | *Fatsia japonica* (Thunb.) Decne. & Planch. | P caesp | Korea, Japan | Subtropical | Neophyte | Casual alien |
| Myrtaceae | *Feijoa sellowiana* (O.Berg) O.Berg | P scap | Brazil, Argentina | Subtropical | Neophyte | Casual alien |
| Asteraceae | *Felicia amelloides* (L.) Voss | H caesp | Cape Province | Subtropical | | Cultivated |
| Cactaceae | *Ferocactus histrix* (DC.) G.E.Linds. | P succ | Mexico | Shrubland | | Cultivated |
| Cactaceae | *Ferocactus latispinus* (Haw.) Britton & Rose | P succ | Mexico | Shrubland | | Cultivated |
| Iridaceae | *Ferraria crispa* Burm. | G bulb | Cape Province | Subtropical | | Cultivated |
| Moraceae | *Ficus altissima* Blume | P scap | China, S-E Asia | Tropical | | Cultivated |
| Moraceae | *Ficus benghalensis* L. | P scap | India | Tropical | | Cultivated |
| Moraceae | *Ficus benjamina* L. | P caesp | S-E Asia, N-E Australia | Tropical | | Cultivated |
| Moraceae | *Ficus benjamina* L. 'Kinky Variegato' | P scap | Horticultural | Tropical | | Cultivated |
| Moraceae | *Ficus carica* L. | P scap | Caucasus | Temperate | | Native |
| Moraceae | *Ficus elastica* Roxb. ex Hornem. | P scap | E Asia | Tropical | Neophyte | Casual alien |

**Table S1.** List of the Sicilian ornamental taxa per Families, Growth Form (according to [41, 42]), Geographical Origin (derived from [41]), Biome Origin (according to [41]), Resident Time and Status (Native/Alien) in Italy (derived both from [43,44]). New records from Sicily are reported with an asterisk.

| FAMILIES | TAXA | GROWTH FORM | GEOGRAPHICAL ORIGIN | BIOME ORIGIN | RESIDENT TIME | STATUS IN ITALY |
|---|---|---|---|---|---|---|
| Moraceae | *Ficus elastica* Roxb. ex Hornem. 'Decora Variegata' | P scap | Horticultural | Tropical | | Cultivated |
| Moraceae | *Ficus elastica* var. *decora* Guillaumin | P scap | E Asia | Tropical | | Cultivated |
| Moraceae | *Ficus heterophylla* L.f. | P scap | E India | Tropical | | Cultivated |
| Moraceae | *Ficus lyrata* Warb. | P scap | C-W Africa | Tropical | | Cultivated |
| Moraceae | *Ficus macrophylla* f. *columnaris* (C.Moore) D.J.Dixon | P scap | Lord Howe Is. | Tropical | Neophyte | Casual alien |
| Moraceae | *Ficus macrophylla* f. *whitakeri* Raimondo & Bajona | P scap | Australia | Tropical | | Cultivated |
| Moraceae | *Ficus macrophylla* Pers. | P scap | E Australia | Tropical | | Cultivated |
| Moraceae | *Ficus microcarpa* L.f. | P scap | S-E Asia | Tropical | Neophyte | Naturalized alien |
| Moraceae | *Ficus pumila* L. | P caesp | S-E Asia | Tropical | Neophyte | Casual alien |
| Moraceae | *Ficus racemosa* L. | P scap | C-S Asia, N Australia | Subtropical | | Cultivated |
| Moraceae | *Ficus rubiginosa* Desf. ex Vent. | P scap | Australia | Subtropical | Neophyte | Casual alien |
| Moraceae | *Ficus virens* Aiton | P scap | S-E Asia, Caroline Is. | Tropical | | Cultivated |
| Moraceae | *Ficus watkinsiana* F.M.Bailey | P scap | Australia | Tropical | Neophyte | Naturalized alien |
| Rosaceae | *Filipendula vulgaris* Moench | P caesp | Europe, N-W Africa, C Asia | Temperate | | Native |
| Malvaceae | *Firmiana simplex* (L.) W.Wight | P scap | E Asia | Temperate | Neophyte | Casual alien |
| Oleaceae | *Fontanesia philliraeoides* Labill. | P caesp | Turkey, Lebanon | Subtropical | Neophyte | Naturalized alien |
| Oleaceae | *Forsythia ×intermedia* Zabel | P scap | Artificial hybrid | Subtropical | Neophyte | Casual alien |
| Oleaceae | *Fraxinus angustifolia* subsp. *oxycarpa* (M.Bieb. ex Willd.) Franco & Rocha Afonso | P scap | S Europe, S Siberia | Temperate | | Native |
| Oleaceae | *Fraxinus angustifolia* Vahl | P scap | Mediterranean | Temperate | | Native |
| Oleaceae | *Fraxinus ornus* L. | P scap | Mediterranean | Temperate | | Native |
| Iridaceae | *Freesia refracta* (Jacq.) Klatt | G bulb | S Africa | Subtropical | | Cultivated |
| Onagraceae | *Fuchsia ×standishii* J.Harrison | P scap | Artificial hybrid | Tropical | | Cultivated |
| Onagraceae | *Fuchsia coccinea* Ait. | P caesp | S America | Tropical | | Cultivated |
| Asparagaceae | *Furcraea selloa* K.Koch | P scap | S America | Tropical | | Cultivated |
| Asparagaceae | *Furcraea selloa* var. *marginata* Trel. | P scap | S America | Tropical | | Cultivated |
| Asteraceae | *Gaillardia aristata* Pursh | H caesp | N America | Temperate | | Cultivated |
| Rubiaceae | *Gardenia jasminoides* J.Ellis | P caesp | China | Subtropical | | Cultivated |
| Rubiaceae | *Gardenia thunbergia* Thunb. | P caesp | S Africa | Subtropical | | Cultivated |
| Aloaceae | *Gasteria carinata* var. *verrucosa* (Mill.) van Jaarsv. | Ch succ | S Africa | Subtropical | | Cultivated |
| Asteraceae | *Gazania rigens* (L.) Gaertn. | H caesp | S Africa | Subtropical | Neophyte | Naturalized alien |
| Fabaceae | *Genista tyrrhena* Vals. | P caesp, P scap | C-S Italy | Subtropical | | Native |
| Ginkgoaceae | *Ginkgo biloba* L. | P scap | China | Temperate | Neophyte | Casual alien |
| Fabaceae | *Gleditsia triacanthos* L. | P scap | N America | Temperate | Neophyte | Naturalized alien |

**Table S1.** List of the Sicilian ornamental taxa per Families, Growth Form (according to [41, 42]), Geographical Origin (derived from [41]), Biome Origin (according to [41]), Resident Time and Status (Native/Alien) in Italy (derived both from [43,44]). New records from Sicily are reported with an asterisk.

| FAMILIES | TAXA | GROWTH FORM | GEOGRAPHICAL ORIGIN | BIOME ORIGIN | RESIDENT TIME | STATUS IN ITALY |
|---|---|---|---|---|---|---|
| Colchicaceae | *Gloriosa superba* L. | G tub | C-S Africa, S Asia | Tropical | | Cultivated |
| Amaranthaceae | *Gomphrena globosa* L. | T scap | C-S America | Tropical | Neophyte | Casual alien |
| Crassulaceae | *Graptopetalum paraguayense* (N.E.Br.) E.Walther | NP succ | N-C America | Shrubland | Neophyte | Casual alien |
| Cupressaceae | *Grevillea juniperina* R.Br. | P scap | Australia | Temperate | | Cultivated |
| Cupressaceae | *Grevillea robusta* A.Cunn. | P scap | Australia | Subtropical | Neophyte | Casual alien |
| Cupressaceae | *Grevillea rosmarinifolia* A.Cunn. | P scap | Australia | Subtropical | | Cultivated |
| Gunneraceae | *Gunnera manicata* Linden ex André* | G rhiz | Brazil | Temperate | | Cultivated |
| Amaryllidaceae | *Haemanthus coccineus* L. | G bulb | S Africa | Subtropical | | Cultivated |
| Stilbaceae | *Halleria lucida* L. | P caesp | S Africa | Tropical | | Cultivated |
| Bignoniaceae | *Handroanthus heptaphyllus* (Vell.) Mattos | P scap | S America | Tropical | Neophyte | Casual alien |
| Fabaceae | *Hardenbergia violacea* (Schneev.) Stearn* | P lian | Australia | Subtropical | | Cultivated |
| Anacardiaceae | *Harpephyllum afrum* Bernh.* | P scap | S Africa | Subtropical | | Cultivated |
| Cactaceae | *Harrisia pomanensis* (F.A.C.Weber ex K.Schum.) Britton & Rose | P succ | Argentina | Shrubland | | Cultivated |
| Aloaceae | *Haworthia fasciata* Haw. | P caesp | S Africa | Subtropical | | Cultivated |
| Araliaceae | *Hedera canariensis* Willd. | P lian | Canary Is. | Temperate | Neophyte | Naturalized alien |
| Araliaceae | *Hedera helix* L. | P lian | Europe, W Asia | Temperate | | Native |
| Asteraceae | *Helianthus annuus* L. | T scap | S America | Temperate | Neophyte | Casual alien |
| Asteraceae | *Helianthus tuberosus* L. | G bulb | N America | Temperate | Neophyte | Invasive alien |
| Asteraceae | *Helichrysum litoreum* Guss. | Ch suffr | S Italy, Sicily | Temperate | | Native |
| Boraginaceae | *Heliotropium arborescens* L. | Ch frut | S America | Tropical | Neophyte | Casual alien |
| Asphodelaceae | *Hemerocallis fulva* (L.) L. | G rhiz | China, E Asia | Temperate | Neophyte | Naturalized alien |
| Asphodelaceae | *Hemerocallis lilioasphodelus* L. | G bulb | S-E Europe, C-E Asia | Temperate | | Native |
| Araliaceae | *Heptapleurum actinophyllum* (Endl.) Lowry & G.M.Plunkett | P caesp | New Guinea, N-E Australia | Tropical | | Cultivated |
| Araliaceae | *Heptapleurum arboricola* Hayata | P caesp, P scap | Taiwan | Tropical | Neophyte | Casual alien |
| Araliaceae | *Heptapleurum divaricatum* (Blume) Seem | P scap | Sumatra, Java | Tropical | | Cultivated |
| Araliaceae | *Heptapleurum heptaphyllum* (L.) Y.F.Deng | P caesp, P scap | S-E Asia | Subtropical | | Cultivated |
| Cupressaceae | *Hesperocyparis arizonica* (Greene) Bartel | P scap | N America | Temperate | Neophyte | Naturalized alien |
| Cupressaceae | *Hesperocyparis benthamii* (Endl.) Bartel | P scap | Mexico | Subtropical | | Cultivated |
| Cupressaceae | *Hesperocyparis lusitanica* (Mill.) Bartel | P scap | C America | Subtropical | | Cultivated |
| Cupressaceae | *Hesperocyparis macrocarpa* (Hartw.) Bartel | P scap | California | Temperate | Neophyte | Naturalized alien |
| Cupressaceae | *×Hesperotropsis leylandii* (A.B.Jacks. & Dallim.) Garland & Gerry Moore | P scap | Artificial hybrid | Temperate | | Cultivated |
| Malvaceae | *Hibiscus ×rosa-sinensis* L. | P caesp | W Pacific | Tropical | Neophyte | Casual alien |
| Malvaceae | *Hibiscus cannabinus* L. | P caesp | C-S Africa | Tropical | | Cultivated |

**Table S1.** List of the Sicilian ornamental taxa per Families, Growth Form (according to [41, 42]), Geographical Origin (derived from [41]), Biome Origin (according to [41]), Resident Time and Status (Native/Alien) in Italy (derived both from [43,44]). New records from Sicily are reported with an asterisk.

| FAMILIES | TAXA | GROWTH FORM | GEOGRAPHICAL ORIGIN | BIOME ORIGIN | RESIDENT TIME | STATUS IN ITALY |
|---|---|---|---|---|---|---|
| Malvaceae | *Hibiscus liliiflorus* Cav. | P caesp | Reunion Is. | Tropical | | Cultivated |
| Malvaceae | *Hibiscus moscheutos* L. | P caesp | N-C America | Temperate | Neophyte | Naturalized alien |
| Malvaceae | *Hibiscus mutabilis* L. | P caesp, P scap | China, Taiwan | Subtropical | | Cultivated |
| Malvaceae | *Hibiscus syriacus* L. | P caesp | E Asia | Temperate | Neophyte | Casual alien |
| Fabaceae | *Hippocrepis emerus* (L.) Lassen | NP | Europe, Caucasus | Temperate | | Cultivated |
| Lamiaceae | *Holmskioldia sanguinea* Retz. | P caesp | C Asia | Tropical | | Cultivated |
| Arecaceae | *Howea belmoreana* (C.Moore & F.Muell.) Becc. | P scap | Australia, Lord Howe Is. | Tropical | | Cultivated |
| Arecaceae | *Howea forsteriana* (F.Muell.) Becc. | P scap | Lord Howe Is. | Tropical | | Cultivated |
| Asclepiadaceae | *Hoya carnosa* (L.) R.Br. | P lian | E Asia | Subtropical | | Cultivated |
| Hyacinthaceae | *Hyacinthus orientalis* L. | G bulb | W Asia | Temperate | Archaeophyte | Casual alien |
| Hydrangeaceae | *Hydrangea macrophylla* (Thunb.) Ser. | P caesp | Japan | Temperate | Neophyte | Naturalized alien |
| Hydrangeaceae | *Hydrangea serrata* (Thunb.) Ser. | P caesp | Korea, Japan | Temperate | | Cultivated |
| Araliaceae | *Hydrocotyle vulgaris* L. | G rhiz, I rad | Europe | Temperate | | Native |
| Aquifoliaceae | *Ilex aquifolium* L. | P caesp, P scap | Europe, N-W Africa | Temperate | | Native |
| Aquifoliaceae | *Ilex aquifolium* L. 'Aureo-marginata' | P scap | Horticultural | Temperate | | Cultivated |
| Solanaceae | *Iochroma cyaneum* (Lindl.) M.L.Green | P scap | S America | Tropical | | Cultivated |
| Solanaceae | *Iochroma gesnerioides* (Kunth) Miers | P scap | C America | Tropical | | Cultivated |
| Convolvulaceae | *Ipomoea alba* L. | G rhiz | C-S America | Tropical | | Cultivated |
| Convolvulaceae | *Ipomoea cairica* (L.) Sweet | G rhiz | C-S Africa, S Asia | Tropical | Neophyte | Naturalized alien |
| Convolvulaceae | *Ipomoea indica* (Burm.) Merr. | G rhiz | C-S America | Tropical | Neophyte | Invasive alien |
| Convolvulaceae | *Ipomoea purpurea* (L.) Roth | P lian | C-S America | Tropical | Neophyte | Naturalized alien |
| Iridaceae | *Iris ×germanica* L. | G rhiz | E Mediterranean | Temperate | Archaeophyte | Naturalized alien |
| Iridaceae | *Iris foetidissima* L. | G rhiz | E Mediterranean | Temperate | | Cultivated |
| Iridaceae | *Iris virginica* L. | G rhiz | USA, Canada | Temperate | | Cultivated |
| Bignoniaceae | *Jacaranda mimosifolia* D.Don | P scap | S America | Subtropical | Neophyte | Casual alien |
| Asteraceae | *Jacobaea maritima* (L.) Pelser & Meijden | Ch suffr | C-W Mediterranean | Subtropical | | Native |
| Oleaceae | *Jasminum azoricum* L. | P caesp | Azores | Temperate | | Cultivated |
| Oleaceae | *Jasminum grandiflorum* L. | P caesp | C-E Africa, C Asia | Subtropical | | Cultivated |
| Oleaceae | *Jasminum mesnyi* Hance | P caesp | China, Vietnam | Subtropical | Neophyte | Naturalized alien |
| Oleaceae | *Jasminum nudiflorum* Lindl. | P caesp | China | Temperate | Neophyte | Naturalized alien |
| Oleaceae | *Jasminum officinale* L. | P caesp | W Asia | Temperate | Archaeophyte | Naturalized alien |
| Oleaceae | *Jasminum polyanthum* Franch. | P caesp | China | Subtropical | Neophyte | Casual alien |
| Oleaceae | *Jasminum sambac* (L.) W.Aiton | P caesp | S-E Asia | Tropical | | Cultivated |

**Table S1.** List of the Sicilian ornamental taxa per Families, Growth Form (according to [41, 42]), Geographical Origin (derived from [41]), Biome Origin (according to [41]), Resident Time and Status (Native/Alien) in Italy (derived both from [43,44]). New records from Sicily are reported with an asterisk.

| FAMILIES | TAXA | GROWTH FORM | GEOGRAPHICAL ORIGIN | BIOME ORIGIN | RESIDENT TIME | STATUS IN ITALY |
|---|---|---|---|---|---|---|
| Oleaceae | *Jasminum simplicifolium* G.Forst. | P caesp | S-E Asia, E Australia | Tropical | | Cultivated |
| Solanaceae | *Juanulloa mexicana* (Schltdl.) Miers | P scap | C-S America | Tropical | | Cultivated |
| Arecaceae | *Jubaea chilensis* (Molina) Baill. | P Scap | Chile | Subtropical | | Cultivated |
| Juglandaceae | *Juglans regia* L. | P scap | Caucasus | Temperate | | Native/Cryptogenic |
| Cupressaceae | *Juniperus chinensis* f. *pfltzeriana* (Spaeth) Rehder | P caesp, P scap | E Asia | Temperate | Neophyte | Casual alien |
| Cupressaceae | *Juniperus chinensis* L. | P caesp, P scap | E Asia | Temperate | Neophyte | Casual alien |
| Cupressaceae | *Juniperus communis* L. | P caesp | Subartic, Northern Hemisphere | Temperate | | Native |
| Cupressaceae | *Juniperus oxycedrus* L. | P caesp | W-C Mediterranean | Temperate | | Native |
| Cupressaceae | *Juniperus virginiana* L. | P caesp | USA | Temperate | Neophyte | Casual alien |
| Acanthaceae | *Justicia adhatoda* L. | P caesp | S-E Asia | Tropical | Neophyte | Doubtful |
| Acanthaceae | *Justicia brandegeeana* Wassh. & L.B.Sm.* | P caesp | C America | Tropical | | Cultivated |
| Acanthaceae | *Justicia carnea* Lindl.* | P caesp | S America | Tropical | | Cultivated |
| Acanthaceae | *Justicia macrantha* Benth. | P caesp | C America | Tropical | | Cultivated |
| Acanthaceae | *Justicia spicigera* Schltdl. | P caesp | C America | Tropical | | Cultivated |
| Crassulaceae | *Kalanchoe beharensis* Drake | P succ | Madagascar | Shrubland | | Cultivated |
| Crassulaceae | *Kalanchoe blossfeldiana* Poelln. | P succ | Madagascar | Tropical | | Cultivated |
| Crassulaceae | *Kalanchoe daigremontiana* Raym.-Hamet & H.Perrier | P succ | Madagascar | Shrubland | Neophyte | Naturalized alien |
| Crassulaceae | *Kalanchoe marmorata* Baker | P succ | C-E Africa | Tropical | | Cultivated |
| Fabaceae | *Kennedia rubicunda* (Schneev.) Vent. | P lian | Australia | Tropical | | Cultivated |
| Rosaceae | *Kerria japonica* (L.) DC. | P caesp | China, Japan | Temperate | Neophyte | Naturalized alien |
| Asteraceae | *Kleinia anteuphorbium* (L.) DC. | P succ | Morocco | Shrubland | Neophyte | Casual alien |
| Asteraceae | *Kleinia neriifolia* Haw. | NP | Canary Is. | Subtropical | Neophyte | Casual alien |
| Asteraceae | *Kleinia stapeliiformis* Stapf | P succ | S Africa | Subtropical | | Cultivated |
| Asphodelaceae | *Kniphofia uvaria* (L.) Oken | Ch succ | Cape Province | Temperate | Neophyte | Casual alien |
| Sapindaceae | *Koelreuteria paniculata* Laxm. | P scap | China, Korea | Temperate | Neophyte | Naturalized alien |
| Cactaceae | *Kroenleinia grusonii* (Hildm.) Lodé | Ch succ | Mexico | Shrubland | | Cultivated |
| Asphodelaceae | *Kumara plicatilis* (L.) G.D.Rowley* | P caesp | S Africa | Subtropical | | Cultivated |
| Fabaceae | *Laburnum anagyroides* Medik. | P caesp | S Europe | Temperate | | Native |
| Lythraceae | *Lagerstroemia indica* L. | P caesp, P scap | S Asia | Subtropical | Neophyte | Casual alien |
| Malvaceae | *Lagunaria patersonia* (Andrews) G.Don | P scap | E Australia | Tropical | Neophyte | Casual alien |
| Aizoaceae | *Lampranthus spectabilis* (Haw.) N.E.Br. | Ch suffr | Cape Province | Subtropical | | Cultivated |
| Verbenaceae | *Lantana camara* L. | P caesp | C-S America | Subtropical | Neophyte | Naturalized alien |
| Verbenaceae | *Lantana camara* L. 'Variegata'* | P caesp | Horticultural | Tropical | | Cultivated |

**Table S1.** List of the Sicilian ornamental taxa per Families, Growth Form (according to [41, 42]), Geographical Origin (derived from [41]), Biome Origin (according to [41]), Resident Time and Status (Native/Alien) in Italy (derived both from [43,44]). New records from Sicily are reported with an asterisk.

| FAMILIES | TAXA | GROWTH FORM | GEOGRAPHICAL ORIGIN | BIOME ORIGIN | RESIDENT TIME | STATUS IN ITALY |
|---|---|---|---|---|---|---|
| Verbenaceae | *Lantana montevidensis* (Spreng.) Briq. | P caesp | S America | Tropical | Neophyte | Casual alien |
| Lauraceae | *Laurus azorica* (Seub.) Franco | P caesp, P scap | Azores | Subtropical | | Cultivated |
| Lauraceae | *Laurus nobilis* L. | P caesp, P scap | Mediterranean | Subtropical | | Native |
| Lamiaceae | *Lavandula angustifolia* Mill. | NP | S-W Europe | Temperate | | Native |
| Lamiaceae | *Lavandula dentata* L. | NP | W Mediterranean, N-E Africa | Subtropical | Neophyte | Casual alien |
| Lamiaceae | *Leonotis leonurus* (L.) R.Br. | P caesp | S Africa | Subtropical | | Cultivated |
| Myrtaceae | *Leptospermum scoparium* J.R.Forst. & G.Forst. | P scap | Australia, New Zeland | Subtropical | | Cultivated |
| Fabaceae | *Leucaena leucocephala* (Lam.) de Wit | P scap | C America | Tropical | | Cultivated |
| Fabaceae | *Leucaena leucocephala* subsp. *glabrata* (Rose) Zárate | P scap | C America | Tropical | Neophyte | Naturalized alien |
| Asteraceae | *Leucanthemum maximum* (Ramond) DC. | H scap | W Europe | Temperate | | Cultivated |
| Cactaceae | *Leucostele atacamensis* subsp. *pasacana* (F.A.C.Weber ex Rümpler) Schlumpb. | P succ | S America | Shrubland | | Cultivated |
| Oleaceae | *Ligustrum japonicum* Thunb. | P caesp, P scap | E Asia | Temperate | Neophyte | Casual alien |
| Oleaceae | *Ligustrum japonicum* var. *aureovariegatum* Veitch ex J.Dix | P caesp, P scap | E Asia | Temperate | Neophyte | Casual alien |
| Oleaceae | *Ligustrum japonicum* var. *rotundifolium* Blume | P caesp, P scap | E Asia | Temperate | Neophyte | Casual alien |
| Oleaceae | *Ligustrum lucidum* W.T.Aiton | P scap | E Asia | Temperate | Neophyte | Invasive alien |
| Oleaceae | *Ligustrum ovalifolium* Hassk. | P caesp, P scap | Japan | Temperate | Neophyte | Invasive alien |
| Oleaceae | *Ligustrum ovalifolium* Hassk. 'Variegatum' | P caesp, P scap | Japan | Temperate | | Cultivated |
| Oleaceae | *Ligustrum sinense* Lour. | P caesp, P scap | S-E Asia | Subtropical | Neophyte | Invasive alien |
| Oleaceae | *Ligustrum vulgare* L. | P caesp | Europe, N Africa, Iran | Temperate | | Native |
| Liliaceae | *Lilium candidum* L. | G bulb | E Mediterranean, W Asia | Temperate | Archaeophyte | Naturalized alien |
| Plumbaginaceae | *Limoniastrum monopetalum* (L.) Boiss. in DC. | Ch frut | S Europa, N Africa | Subtropical | | Native |
| Plumbaginaceae | *Limonium sinuatum* (L.) Mill. | H scap | Mediterranean | Temperate | | Native |
| Altingiaceae | *Liquidambar styraciflua* L.* | P scap | USA, C America | Temperate | Neophyte | Casual alien |
| Arecaceae | *Livistona australis* (R.Br.) Mart. | P scap | S-E Australia | Subtropical | | Cultivated |
| Arecaceae | *Livistona chinensis* (Jacq.) R.Br. ex Mart. | P scap | Japan, China | Subtropical | | Cultivated |
| Arecaceae | *Livistona decora* (W.Bull) Dowe | P scap | Queensland | Tropical | | Cultivated |
| Caprifoliaceae | *Lonicera implexa* Aiton | P lian | Mediterranean | Temperate | | Native |
| Caprifoliaceae | *Lonicera japonica* Thunb. | P caesp | E Asia | Temperate | Neophyte | Invasive alien |
| Caprifoliaceae | *Lonicera ligustrina* var. *pileata* (Oliv.) Franch. | P caesp | China | Temperate | Neophyte | Naturalized alien |
| Caprifoliaceae | *Lonicera periclymenum* L. | P lian | Europe, Morocco | Temperate | | Native |
| Cactaceae | *Lophocereus marginatus* (DC.) S.Arias & Terrazas | P succ | Mexico | Shrubland | | Cultivated |
| Hamamelidaceae | *Loropetalum chinense* (R.Br.) Oliv.* | P caesp | China, Japan | Temperate | | Cultivated |
| Solanaceae | *Lycianthes lycioides* (L.) Hassl. | NP | S America | Tropical | | Cultivated |

**Table S1.** List of the Sicilian ornamental taxa per Families, Growth Form (according to [41, 42]), Geographical Origin (derived from [41]), Biome Origin (according to [41]), Resident Time and Status (Native/Alien) in Italy (derived both from [43,44]). New records from Sicily are reported with an asterisk.

| FAMILIES | TAXA | GROWTH FORM | GEOGRAPHICAL ORIGIN | BIOME ORIGIN | RESIDENT TIME | STATUS IN ITALY |
|---|---|---|---|---|---|---|
| Solanaceae | *Lycianthes rantonnetii* (Carrière) Bitter | NP | S America | Subtropical | Neophyte | Naturalized alien |
| Solanaceae | *Lycium barbarum* L. | NP | China | Temperate | Neophyte | Naturalized alien |
| Fabaceae | *Lysiphyllum diphyllum* (Banks) de Wit | P scap | S-E Asia | Tropical | | Cultivated |
| Moraceae | *Maclura pomifera* (Rafin.) C.K.Schneider | P scap | USA | Temperate | Neophyte | Naturalized alien |
| Magnoliaceae | *Magnolia ×soulangeana* Soul.-Bod. | P scap | China | Temperate | | Cultivated |
| Magnoliaceae | *Magnolia grandiflora* L. | P scap | N America | Subtropical | Neophyte | Casual alien |
| Aizoaceae | *Malephora crocea* (Jacq.) Schwantes | Ch succ | Cape Province | Shrubland | Neophyte | Invasive alien |
| Rosaceae | *Malus domestica* (Suckow) Borkh. | P scap | C Asia | Temperate | Archaeophyte | Naturalized alien |
| Malvaceae | *Malva olbia* (L.) Alef. | P caesp | W-C Mediterranean | Subtropical | | Native |
| Malvaceae | *Malvaviscus arboreus* Dill. ex Cav. | P scap | C-S America | Tropical | | Cultivated |
| Malvaceae | *Malvaviscus arboreus* var. *arboreus* | P scap | Mexico | Tropical | | Cultivated |
| Apocynaceae | *Mandevilla laxa* (Ruiz & Pav.) Woodson | P lian | S America | Tropical | | Cultivated |
| Apocynaceae | *Mandevilla sanderi* (Hemsl.) Woodson | P lian | S America | Tropical | | Cultivated |
| Anacardiaceae | *Mangifera indica* L. | P scap | E India, Malesia | Tropical | | Cultivated |
| Brassicaceae | *Matthiola incana* (L.) W.T.Aiton | Ch suffr | S-W Europe | Temperate | | Native |
| Fabaceae | *Medicago arborea* L. | P caesp | N Mediterranean | Subtropical | | Native |
| Myrtaceae | *Melaleuca citrina* (Curtis) Dum.Cours. | P caesp | Australia | Subtropical | | Cultivated |
| Myrtaceae | *Melaleuca rugulosa* (Link) Craven | P caesp | Australia | Subtropical | | Cultivated |
| Myrtaceae | *Melaleuca thymifolia* Sm. | P caesp, P scap | Queensland | Subtropical | | Cultivated |
| Myrtaceae | *Melaleuca viminalis* (Sol. ex Gaertn.) Byrnes | P caesp | Australia | Subtropical | | Cultivated |
| Meliaceae | *Melia azedarach* L. | P scap | E Asia, Australia | Tropical | Neophyte | Naturalized alien |
| Araliaceae | *Meryta denhamii* Seem. | P scap | New Caledonia | Tropical | | Cultivated |
| Aizoaceae | *Mesembryanthemum cordifolium* L.f. | Ch suffr | Cape Province | Subtropical | Neophyte | Invasive alien |
| Myrtaceae | *Metrosideros excelsa* Sol. ex Gaertn. | P scap | New Zealand | Subtropical | Neophyte | Casual alien |
| Myrtaceae | *Metrosideros robusta* A.Cunn. | P scap | New Zealand | Subtropical | | Cultivated |
| Nyctaginaceae | *Mirabilis jalapa* L. | G bulb | C America | Subtropical | Neophyte | Invasive alien |
| Araceae | *Monstera deliciosa* Liebm. | P lian | C America | Tropical | | Cultivated |
| Asteraceae | *Montanoa bipinnatifida* (Kunth) K.Koch | P caesp | Mexico | Subtropical | Neophyte | Casual alien |
| Moraceae | *Morus alba* L. | P scap | E Asia | Temperate | Archaeophyte | Naturalized alien |
| Moraceae | *Morus alba* L. 'Pendula' | P scap | Horticultural | Temperate | | Cultivated |
| Moraceae | *Morus nigra* L. | P scap | W Asia | Temperate | Archaeophyte | Naturalized alien |
| Polygonaceae | *Muehlenbeckia complexa* (A.Cunn.) Meisn. | NP | Lord Howe Is., New Zealand | Temperate | | Cultivated |
| Polygonaceae | *Muehlenbeckia platyclada* (F.Muell.) Meisn. | NP | Papuasia | Tropical | | Cultivated |

**Table S1.** List of the Sicilian ornamental taxa per Families, Growth Form (according to [41, 42]), Geographical Origin (derived from [41]), Biome Origin (according to [41]), Resident Time and Status (Native/Alien) in Italy (derived both from [43,44]). New records from Sicily are reported with an asterisk.

| FAMILIES | TAXA | GROWTH FORM | GEOGRAPHICAL ORIGIN | BIOME ORIGIN | RESIDENT TIME | STATUS IN ITALY |
|---|---|---|---|---|---|---|
| Rutaceae | *Murraya paniculata* (L.) Jack | P scap | S-E Asia | Tropical | | Cultivated |
| Musaceae | *Musa ×paradisiaca* L. | G rhiz | Malesia | Tropical | Neophyte | Casual alien |
| Scrophulariaceae | *Myoporum insulare* R.Br. | P caesp, P scap | Australia | Subtropical | Neophyte | Invasive alien |
| Scrophulariaceae | *Myoporum tenuifolium* G.Forst. | P caesp, P scap | New Caledonia | Subtropical | | Cultivated |
| Myrtaceae | *Myrtus communis* L. | P caesp, P scap | Mediterranean, W Asia | Temperate | | Native |
| Myrtaceae | *Myrtus communis* subsp. *tarentina* (L.) Nyman | P caesp, P scap | S Europe | Temperate | | Native |
| Berberidaceae | *Nandina domestica* Thunb. | P caesp | China | Temperate | Neophyte | Casual alien |
| Amaryllidaceae | *Narcissus ×incomparabilis* Mill. | G bulb | France | Temperate | | Cultivated |
| Amaryllidaceae | *Narcissus tazetta* L. | G bulb | Mediterranean | Subtropical | | Native |
| Nelumbonaceae | *Nelumbo nucifera* Gaertn. | I rad | S Asia | Subtropical | Neophyte | Invasive alien |
| Polypodiaceae | *Nephrolepis cordifolia* (L.) C.Presl | H caesp | C-W Africa, S-E Asia, E Australia | Subtropical | Neophyte | Naturalized alien |
| Apocynaceae | *Nerium oleander* L. | P caesp | Mediterranean, S-W Asia | Subtropical | | Native |
| Apocynaceae | *Nerium oleander* var. *luteomarginatum* Van Geert | P caesp | Mediterranean, S-W Asia | Subtropical | | Native |
| Solanaceae | *Nicotiana glauca* Graham | P caesp, P scap | S America | Subtropical | Neophyte | Invasive alien |
| Asparagaceae | *Nolina parviflora* (Kunth) Hemsl. | P caesp | Mexico | Tropical | | Cultivated |
| Nymphaeaceae | *Nuphar lutea* (L.) Sm. | I rad | Europe, N-C Asia | Temperate | | Native |
| Nymphaeaceae | *Nymphaea alba* L. | I rad | Europe, N Africa, N-C Asia | Temperate | | Native |
| Nymphaeaceae | *Nymphaea odorata* subsp. *tuberosa* (Paine) Wiersema & Hellq. | I rad | N America | Temperate | | Cultivated |
| Lamiaceae | *Ocimum basilicum* L. | T scap | S-E Asia, Australia | Shrubland | Archaeophyte | Casual alien |
| Onagraceae | *Oenothera lindheimeri* (Engelm. & A.Gray) W.L.Wagner & Hoch* | H scap | USA | Temperate | Neophyte | Naturalized alien |
| Onagraceae | *Oenothera stricta* Ledeb. ex Link | H scap | S America | Temperate | Neophyte | Naturalized alien |
| Oleaceae | *Olea europaea* L. | P scap | Mediterranean, Africa, S-W Asia | Temperate | | Native |
| Cactaceae | *Opuntia dejecta* Salm-Dyck | P succ | C America | Tropical | Neophyte | Naturalized alien |
| Cactaceae | *Opuntia engelmannii* var. *lindheimeri* (Engelm.) B.D.Parfitt & Pinkava | P succ | USA, Mexico | Shrubland | Neophyte | Naturalized alien |
| Cactaceae | *Opuntia erinacea* var. *hystricina* (Engelm. & J.M.Bigelow) L.D.Benson | P succ | Arizona | Shrubland | | Cultivated |
| Cactaceae | *Opuntia ficus-indica* (L.) Mill. | P succ | Mexico | Tropical | Neophyte | Invasive alien |
| Cactaceae | *Opuntia leucotricha* DC. | P succ | Mexico | Shrubland | Neophyte | Naturalized alien |
| Cactaceae | *Opuntia maxima* Mill. | P succ | Mexico | Shrubland | | Cultivated |
| Cactaceae | *Opuntia microdasys* (Lehm.) Pfeiff. | P succ | Mexico | Shrubland | Neophyte | Naturalized alien |
| Cactaceae | *Opuntia monacantha* Haw. | P succ | S America | Tropical | Neophyte | Naturalized alien |
| Cactaceae | *Opuntia phaeacantha* Engelm. | P succ | USA, Mexico | Temperate | Neophyte | Invasive alien |
| Cactaceae | *Opuntia robusta* H.L.Wendl. ex Pfeiff. | P succ | Mexico | Shrubland | Neophyte | Naturalized alien |
| Cactaceae | *Opuntia streptacantha* Lem. | P succ | C America | Tropical | Neophyte | Naturalized alien |

**Table S1.** List of the Sicilian ornamental taxa per Families, Growth Form (according to [41, 42]), Geographical Origin (derived from [41]), Biome Origin (according to [41]), Resident Time and Status (Native/Alien) in Italy (derived both from [43,44]). New records from Sicily are reported with an asterisk.

| FAMILIES | TAXA | GROWTH FORM | GEOGRAPHICAL ORIGIN | BIOME ORIGIN | RESIDENT TIME | STATUS IN ITALY |
|---|---|---|---|---|---|---|
| Cactaceae | *Opuntia stricta* (Haw.) Haw. | P succ | USA, Mexico | Shrubland | Neophyte | Invasive alien |
| Cactaceae | *Opuntia tomentosa* Salm-Dyck | P succ | C America | Shrubland | Neophyte | Naturalized alien |
| Cactaceae | *Opuntia tuna* (L.) Mill. | P succ | C America | Tropical | Neophyte | Naturalized alien |
| Araliaceae | *Oreopanax dactylifolius* T.Moore | P caesp, P scap | Mexico | Tropical | | Cultivated |
| Araliaceae | *Oreopanax nymphaeifolius* (Hibberd) Decne. & Planch. ex G.Nicholson | P scap | C America | Tropical | | Cultivated |
| Oleaceae | *Osmanthus fragrans* Lour. | P caesp, P scap | C-E Asia | Subtropical | | Cultivated |
| Oleaceae | *Osmanthus heterophyllus* (G.Don) P.S.Green* | P caesp, P scap | Korea, Japan | Temperate | | Cultivated |
| Rosaceae | *Osteomeles schweriniae* C.K.Schneid. | P scap | China | Temperate | | Cultivated |
| Malvaceae | *Pachira aquatica* Aubl.* | P scap | C-S America | Tropical | | Cultivated |
| Menispermaceae | *Pachygone laurifolia* (DC.) L.Lian & Wei Wang | P scap | S-E Asia | Tropical | Neophyte | Naturalized alien |
| Apocynaceae | *Pachypodium lamerei* Drake* | P succ | Madagascar | Tropical | | Cultivated |
| Paeoniaceae | *Paeonia ×suffruticosa* Andrews | Ch suffr | China | Temperate | Neophyte | Historical record |
| Rhamnaceae | *Paliurus spina-christi* Mill. | P caesp | Mediterranean, W Asia | Subtropical | | Native |
| Amaryllidaceae | *Pancratium maritimum* L. | G bulb | Mediterranean | Subtropical | | Native |
| Bignoniaceae | *Pandorea jasminoides* (Lindl.) K.Schum. | P lian | Australia | Subtropical | | Cultivated |
| Fabaceae | *Pararchidendron pruinosum* (Benth.) I.C.Nielsen | P caesp, P scap | Malaysia, New Guinea, E Australia | Subtropical | | Cultivated |
| Fabaceae | *Parasenegalia visco* (Lorentz ex Griseb.) Seigler & Ebinger | P scap | S America | Subtropical | Neophyte | Naturalized alien |
| Fabaceae | *Paraserianthes lophantha* (Vent.) I.C.Nielsen | P scap | S-E Asia, Australia | Subtropical | Neophyte | Naturalized alien |
| Fabaceae | *Parkinsonia aculeata* L. | P scap | C-S America | Tropical | Neophyte | Invasive alien |
| Vitaceae | *Parthenocissus quinquefolia* (L.) Planch. | P lian | N America | Temperate | Neophyte | Invasive alien |
| Vitaceae | *Parthenocissus tricuspidata* (Siebold & Zucc.) Planch.* | P lian | E Asia | Temperate | Neophyte | Naturalized alien |
| Passifloraceae | *Passiflora caerulea* L. | P lian | S America | Subtropical | Neophyte | Naturalized alien |
| Paulowniaceae | *Paulownia tomentosa* (Thunb.) Steud. | P scap | China, Korea | Temperate | Neophyte | Invasive alien |
| Geraniaceae | *Pelargonium capitatum* (L.) L'Hér. | Ch suffr | Cape Province | Subtropical | Neophyte | Casual alien |
| Geraniaceae | *Pelargonium graveolens* L'Hér. | Ch suffr | S Africa | Subtropical | Neophyte | Casual alien |
| Geraniaceae | *Pelargonium peltatum* (L.) L'Hér. | Ch suffr | Cape Province | Subtropical | Neophyte | Casual alien |
| Geraniaceae | *Pelargonium zonale* (L.) L'Hér. | Ch suffr | Cape Province | Subtropical | | Cultivated |
| Piperaceae | *Peperomia magnoliifolia* (Jacq.) A.Dietr. | Ch succ | C-S America | Tropical | | Cultivated |
| Piperaceae | *Peperomia obtusifolia* (L.) A.Dietr. | Ch succ | C-S America | Tropical | | Cultivated |
| Lauraceae | *Persea americana* Mill. | P scap | C America | Tropical | Neophyte | Casual alien |
| Lauraceae | *Persea indica* (L.) Spreng. | P scap | Canary Is. | Subtropical | Neophyte | Historical record |
| Crassulaceae | *Petrosedum rupestre* (L.) P.V.Heath | Ch succ | C-W Europe, Turkey | Temperate | | Native |
| Crassulaceae | *Petrosedum sediforme* (Jacq.) Grulich | Ch succ | Mediterranean | Subtropical | | Native |

**Table S1.** List of the Sicilian ornamental taxa per Families, Growth Form (according to [41, 42]), Geographical Origin (derived from [41]), Biome Origin (according to [41]), Resident Time and Status (Native/Alien) in Italy (derived both from [43,44]). New records from Sicily are reported with an asterisk.

| FAMILIES | TAXA | GROWTH FORM | GEOGRAPHICAL ORIGIN | BIOME ORIGIN | RESIDENT TIME | STATUS IN ITALY |
|---|---|---|---|---|---|---|
| Solanaceae | *Petunia ×atkinsiana* (Sweet) D.Don ex W.H.Baxter | T scap | Artificial hybrid | Subtropical | Neophyte | Naturalized alien |
| Hydrangeaceae | *Philadelphus coronarius* L. | P caesp | Caucasus | Temperate | | Native |
| Oleaceae | *Phillyrea latifolia* L. | P caesp | Mediterranean | Temperate | | Native |
| Lamiaceae | *Phlomis fruticosa* L.* | P caesp | E Mediterranean | Subtropical | | Native |
| Arecaceae | *Phoenix canariensis* H.Wildpret | P scap | Canary Is. | Subtropical | Neophyte | Naturalized alien |
| Arecaceae | *Phoenix dactylifera* L. | P scap | Arabian Peninsula, S-W Asia | Subtropical | Archaeophyte | Casual alien |
| Arecaceae | *Phoenix loureiroi* Kunth | P scap | S-E Asia | Tropical | | Cultivated |
| Arecaceae | *Phoenix reclinata* Jacq. | P scap | C-S Africa | Tropical | | Cultivated |
| Arecaceae | *Phoenix roebelenii* O'Brien | P caesp | China | Subtropical | Neophyte | Casual alien |
| Arecaceae | *Phoenix rupicola* T.Anderson | P scap | Himalaya, Bangladesh | Subtropical | | Cultivated |
| Asphodelaceae | *Phormium tenax* J.R.Forst. & G.Forst. | G rhiz | Norfolk Is., New Zealand | Temperate | Neophyte | Casual alien |
| Asphodelaceae | *Phormium tenax* J.R.Forst. & G.Forst. 'Variegata'* | G rhiz | Horticultural | Temperate | | Cultivated |
| Rosaceae | *Photinia ×fraseri* Dress | P caesp, P scap | China | Temperate | | Cultivated |
| Rosaceae | *Photinia davidiana* (Decne.) Cardot | P caesp, P scap | S-E Asia | Temperate | | Cultivated |
| Rosaceae | *Photinia glabra* (Thunb.) Pépin | P caesp, P scap | E Asia | Temperate | | Cultivated |
| Rosaceae | *Photinia serratifolia* (Desf.) Kalkman | P caesp, P scap | E Asia | Temperate | Neophyte | Casual alien |
| Poaceae | *Phyllostachys aurea* (André) Rivière & C.Rivière | P caesp | S-E Asia | Temperate | Neophyte | Naturalized alien |
| Poaceae | *Phyllostachys nigra* (Lodd. ex Lindl.) Munro | P caesp | China | Temperate | Neophyte | Naturalized alien |
| Phytolaccaceae | *Phytolacca americana* L. | P scap | N-C America | Temperate | Neophyte | Invasive alien |
| Phytolaccaceae | *Phytolacca dioica* L. | P scap | S America | Tropical | Neophyte | Naturalized alien |
| Pinaceae | *Picea abies* (L.) H.Karst. | P scap | Europe, N-W Asia | Temperate | | Native |
| Pinaceae | *Picea pungens* Engelm. | P scap | N America | Temperate | | Cultivated |
| Pinaceae | *Picea pungens* Engelm. 'Glauca' | P scap | N America | Temperate | | Cultivated |
| Pinaceae | *Picea smithiana* (Willd.) Boiss. | P scap | C Asia | Temperate | | Cultivated |
| Pinaceae | *Pinus banksiana* Lamb. | P scap | N America | Temperate | | Cultivated |
| Pinaceae | *Pinus canariensis* C.Sm. ex DC. | P scap | Canary Is. | Temperate | Neophyte | Naturalized alien |
| Pinaceae | *Pinus halepensis* Mill. | P scap | Mediterranean | Temperate | | Native |
| Pinaceae | *Pinus nigra* J.F.Arnold | P scap | S-E Europe, Caucasus | Temperate | | Native |
| Pinaceae | *Pinus nigra* subsp. *laricio* Palib. ex Maire | P scap | Corse, Italy | Temperate | | Native |
| Pinaceae | *Pinus pinaster* Aiton | P scap | W-C Mediterranean | Temperate | | Native |
| Pinaceae | *Pinus pinea* L. | P scap | S Europe, Lebanon | Temperate | Archaeophyte | Naturalized alien |
| Pinaceae | *Pinus roxburghii* Sarg. | P scap | C Asia | Temperate | | Cultivated |
| Pinaceae | *Pinus wallichiana* A.B.Jacks. | P scap | C Asia | Temperate | Neophyte | Casual alien |

**Table S1.** List of the Sicilian ornamental taxa per Families, Growth Form (according to [41, 42]), Geographical Origin (derived from [41]), Biome Origin (according to [41]), Resident Time and Status (Native/Alien) in Italy (derived both from [43,44]). New records from Sicily are reported with an asterisk.

| FAMILIES | TAXA | GROWTH FORM | GEOGRAPHICAL ORIGIN | BIOME ORIGIN | RESIDENT TIME | STATUS IN ITALY |
|---|---|---|---|---|---|---|
| Nyctagynaceae | *Pisoniella arborescens* (Lag. & Rodr.) Standl. | P lian | S America | Tropical | | Cultivated |
| Anacardiaceae | *Pistacia atlantica* Desf. | P scap | E Mediterranean, E Africa, C Asia | Subtropical | | Cultivated |
| Anacardiaceae | *Pistacia lentiscus* L. | P caesp, P scap | Mediterranean | Subtropical | | Native |
| Anacardiaceae | *Pistacia terebinthus* L. | P caesp, P scap | Mediterranean | Subtropical | | Native |
| Pittosporaceae | *Pittosporum taitense* Putt. | P scap | Society Is. | Tropical | | Cultivated |
| Pittosporaceae | *Pittosporum tenuifolium* Gaertn. 'Silver Queen'* | P scap | Horticultural | Subtropical | | Cultivated |
| Pittosporaceae | *Pittosporum tobira* (Thunb.) W.T.Aiton | P caesp | Korea, Japan | Subtropical | Neophyte | Naturalized alien |
| Pittosporaceae | *Pittosporum tobira* (Thunb.) W.T.Aiton 'Albomarginata' | P caesp | Korea, Japan | Subtropical | | Cultivated |
| Pittosporaceae | *Pittosporum tobira* (Thunb.) W.T.Aiton 'Nanum'* | P caesp | Horticultural | Subtropical | | Cultivated |
| Pittosporaceae | *Pittosporum undulatum* Vent. | P scap | E Australia | Subtropical | Neophyte | Naturalized alien |
| Platanaceae | *Platanus ×hispanica* Mill. ex Münchh. | P scap | Artificial hybrid | Temperate | Neophyte | Invasive alien |
| Platanaceae | *Platanus orientalis* L. | P scap | S-E Europe, Caucasus | Temperate | | Native/Cryptogenic |
| Cupressaceae | *Platycladus orientalis* (L.) Franco | P scap | C-E Asia | Temperate | Neophyte | Naturalized alien |
| Araliaceae | *Plerandra elegantissima* (H.J.Veitch ex Mast.) Lowry, G.M.Plunkett & Frodin | P scap | New Caledonia | Tropical | | Cultivated |
| Plumbaginaceae | *Plumbago auriculata* Lam. | P lian | S Africa | Subtropical | Neophyte | Naturalized alien |
| Plumbaginaceae | *Plumbago auriculata* Lam. 'Alba' | P scap | Horticultural | Subtropical | | Cultivated |
| Apocynaceae | *Plumeria obtusa* L.* | P scap | Mexico, Honduras | Tropical | | Cultivated |
| Apocynaceae | *Plumeria rubra* L. | P scap | C-S America | Tropical | | Cultivated |
| Podocarpaceae | *Podocarpus neriifolius* D.Don | P scap | S-E Asia | Tropical | | Cultivated |
| Bignoniaceae | *Podranea ricasoliana* (Tanfani) Sprague | P lian | S Africa | Subtropical | | Cultivated |
| Polygonaceae | *Polygala myrtifolia* L. | NP | S Africa | Subtropical | Neophyte | Casual alien |
| Pontederiaceae | *Pontederia crassipes* Mart. | I nat | S America | Tropical | Neophyte | Invasive alien |
| Salicaceae | *Populus ×canadensis* Moench | P scap | Artificial hybrid | Temperate | Neophyte | Invasive alien |
| Salicaceae | *Populus ×canescens* (Aiton) Sm. | P scap | Europe, China | Temperate | | Cultivated |
| Salicaceae | *Populus alba* L. | P scap | C-S Europe, C Asia | Temperate | | Native |
| Salicaceae | *Populus alba* L. 'Pyramidalis'* | P scap | Horticultural | Temperate | | Cultivated |
| Salicaceae | *Populus nigra* f. *italica* (Münchh.) A.Andersen | P scap | N Italy | Temperate | | Native |
| Salicaceae | *Populus nigra* L. | P scap | Europe, N Africa, W Asia | Temperate | | Native |
| Portulacaceae | *Portulaca grandiflora* Hook. | T scap | S America | Subtropical | Neophyte | Naturalized alien |
| Portulacaceae | *Portulacaria afra* Jacq. | P succ | S Africa | Subtropical | Neophyte | Casual alien |
| Primulaceae | *Primula vulgaris* Huds. | H ros | Europe, N-W Africa, Caucasus | Temperate | | Cultivated |
| Podocarpaceae | *Prumnopitys andina* (Poepp. ex Endl.) de Laub. | P scap | S America | Temperate | | Cultivated |
| Rosaceae | *Prunus amygdalus* Batsch | P scap | Caucasus | Temperate | | Cultivated |

**Table S1.** List of the Sicilian ornamental taxa per Families, Growth Form (according to [41, 42]), Geographical Origin (derived from [41]), Biome Origin (according to [41]), Resident Time and Status (Native/Alien) in Italy (derived both from [43,44]). New records from Sicily are reported with an asterisk.

| FAMILIES | TAXA | GROWTH FORM | GEOGRAPHICAL ORIGIN | BIOME ORIGIN | RESIDENT TIME | STATUS IN ITALY |
|---|---|---|---|---|---|---|
| Rosaceae | *Prunus armeniaca* L. | P scap | C Asia | Temperate | Archaeophyte | Casual alien |
| Rosaceae | *Prunus avium* (L.) L. | P scap | Europe, N Africa, Caucasus | Temperate | | Native |
| Rosaceae | *Prunus cerasifera* Ehrh. 'Pissardi' | P scap | Horticultural | Temperate | | Naturalized alien |
| Rosaceae | *Prunus domestica* L. | P scap | Caucasus | Temperate | Archaeophyte | Naturalized alien |
| Rosaceae | *Prunus dulcis* (Mill.) D.A.Webb | P scap | Caucasus | Temperate | Archaeophyte | Naturalized alien |
| Rosaceae | *Prunus laurocerasus* L. | P scap | S-E Europe, Caucasus | Temperate | Neophyte | Invasive alien |
| Rosaceae | *Prunus mahaleb* L. | P scap | Europe, N-W Africa, W Asia | Temperate | | Native |
| Rosaceae | *Prunus persica* (L.) Batsch | P caesp, P scap | China | Temperate | Archaeophyte | Naturalized alien |
| Poaceae | *Pseudosasa japonica* (Siebold & Zucc. ex Steud.) Makino ex Nakai | P caesp | Korea, Japan | Temperate | Neophyte | Naturalized alien |
| Lythraceae | *Punica granatum* L. | P scap | Caucasus | Temperate | Archaeophyte | Naturalized alien |
| Lythraceae | *Punica granatum* L. 'Nana' | P caesp | Horticultural | Temperate | | Cultivated |
| Bromeliaceae | *Puya ×berteroniana* Mez | P caesp | Chile | Subtropical | | Cultivated |
| Rosaceae | *Pyracantha angustifolia* (Franch.) C.K.Schneid. | P caesp | C Asia | Temperate | Neophyte | Casual alien |
| Rosaceae | *Pyracantha coccinea* M.Roem. | P caesp | C-S Europe, Caucasus | Temperate | | Native |
| Bignoniaceae | *Pyrostegia venusta* (Ker Gawl.) Miers* | P caesp | C-S America | Tropical | | Cultivated |
| Rosaceae | *Pyrus communis* L. | P scap | Europe, W Asia | Temperate | Archaeophyte | Casual alien |
| Rosaceae | *Pyrus spinosa* Forssk. | P scap | Mediterranean | Temperate | | Native |
| Fagaceae | *Quercus ilex* L. | P scap | S Europe, Mediterranean | Temperate | | Native |
| Fagaceae | *Quercus polymorpha* Schltdl. & Cham. | P scap | C America | Tropical | | Cultivated |
| Fagaceae | *Quercus pubescens* Willd. | P scap | C-W Mediterranean | Temperate | | Cultivated |
| Fagaceae | *Quercus suber* L. | P scap | C-W Mediterranean | Temperate | | Native |
| Fabaceae | *Retama monosperma* (L.) Boiss. | P caesp | W Europe, W Africa, E Mediterranean | Temperate | | Cultivated |
| Rhamnaceae | *Rhamnus alaternus* L. | P caesp | Mediterranean | Temperate | | Native |
| Rosaceae | *Rhaphiolepis indica* (L.) Lindl. | P caesp, P scap | China, Japan | Subtropical | | Cultivated |
| Rosaceae | *Rhaphiolepis umbellata* (Thunb.) Makino | P caesp | E Asia | Subtropical | Neophyte | Casual alien |
| Arecaceae | *Rhapis excelsa* (Thunb.) A.Henry* | P caesp, P scap | China | Tropical | | Cultivated |
| Ericaceae | *Rhododendron simsii* Planch. | P caesp | China, Taiwan | Subtropical | | Cultivated |
| Anacardiaceae | *Rhus coriaria* L. | P caesp, P scap | S Europe, N-W Africa, Caucasus | Subtropical | | Native/Cryptogenic |
| Anacardiaceae | *Rhus typhina* L. | P scap | N America | Temperate | Neophyte | Naturalized alien |
| Euphorbiaceae | *Ricinus communis* L. | P scap | N-E Africa, S Europe | Tropical | Archaeophyte | Invasive alien |
| Fabaceae | *Robinia hispida* L. | P caesp, P scap | USA | Temperate | Neophyte | Naturalized alien |
| Fabaceae | *Robinia neomexicana* A.Gray* | P scap | C America | Temperate | Neophyte | Casual alien |
| Fabaceae | *Robinia pseudoacacia* L. | P scap | N America | Temperate | Neophyte | Invasive alien |

**Table S1.** List of the Sicilian ornamental taxa per Families, Growth Form (according to [41, 42]), Geographical Origin (derived from [41]), Biome Origin (according to [41]), Resident Time and Status (Native/Alien) in Italy (derived both from [43,44]). New records from Sicily are reported with an asterisk.

| FAMILIES | TAXA | GROWTH FORM | GEOGRAPHICAL ORIGIN | BIOME ORIGIN | RESIDENT TIME | STATUS IN ITALY |
|---|---|---|---|---|---|---|
| Fabaceae | *Robinia pseudoacacia* L. 'Umbraculifera'* | P scap | Horticultural | Temperate | | Cultivated |
| Asteraceae | *Roldana petasitis* (Sims) H.Rob. & Brettell | P caesp | Mexico | Subtropical | Neophyte | Naturalized alien |
| Rosaceae | *Rosa ×centifolia* L. | NP | Artificial hybrid | Temperate | Archaeophyte | Casual alien |
| Rosaceae | *Rosa banksiae* W.T.Aiton | NP | China | Temperate | Neophyte | Naturalized alien |
| Rosaceae | *Rosa gallica* L. | NP | C-S Europe, Caucasus | Temperate | | Native |
| Rosaceae | *Rosa indica* L. | NP | China, Taiwan | Temperate | | Cultivated |
| Rosaceae | *Rosa multiflora* Thunb. | NP | Korea, Japan | Temperate | Neophyte | Invasive alien |
| Arecaceae | *Roystonea regia* (Kunth) O.F.Cook* | P scap | C America | Tropical | | Cultivated |
| Acanthaceae | *Ruellia simplex* C.Wright | NP | C-S America | Subtropical | Neophyte | Casual alien |
| Polygonaceae | *Rumex lunaria* L. | NP | Canary Is. | Subtropical | Neophyte | Naturalized alien |
| Asparagaceae | *Ruscus aculeatus* L. | G rhiz | C-S Europe, N Africa, Caucasus | Temperate | | Native |
| Asparagaceae | *Ruscus hypoglossum* L. | G rhiz | S-E Europe, Caucasus | Temperate | | Native |
| Asparagaceae | *Ruscus hypophyllum* L. | G rhiz | S-W Europe, N-W Africa | Subtropical | | Native/Cryptogenic |
| Plantaginaceae | *Russelia equisetiformis* Schltdl. & Cham. | Ch frut | Mexico | Tropical | Neophyte | Casual alien |
| Arecaceae | *Sabal bermudana* L.H.Bailey | P scap | Bermuda | Tropical | | Cultivated |
| Arecaceae | *Sabal mauritiiformis* (H.Karst.) Griseb. & H.Wendl. | P scap | C-S America | Tropical | | Cultivated |
| Arecaceae | *Sabal minor* (Jacq.) Pers. | P caesp | USA, Mexico | Temperate | | Cultivated |
| Arecaceae | *Sabal palmetto* (Walter) Lodd. ex Schult. & Schult.f. | P scap | S-E USA, C America | Subtropical | | Cultivated |
| Poaceae | *Saccharum officinarum* L.* | G rhiz | New Guinea | Tropical | Archaeophyte | Casual alien |
| Salicaceae | *Salix ×pendulina* nothof. *tristis* (Gaudin) I.V.Belyaeva | P caesp | E Asia | Temperate | Neophyte | Casual alien |
| Salicaceae | *Salix alba* L. | P scap | Europe, N-W Africa, C-W Asia | Temperate | | Native |
| Salicaceae | *Salix babylonica* L. | P scap | China, Korea | Temperate | Neophyte | Casual alien |
| Salicaceae | *Salix caprea* L. | P caesp | Europe, W Asia | Temperate | | Native |
| Salicaceae | *Salix pedicellata* Desf. | P scap | S-W Europe, N-W Africa, E Europe | Temperate | | Native |
| Lamiaceae | *Salvia argentea* L.* | H scap | Mediterranean | Temperate | | Native |
| Lamiaceae | *Salvia chamaedryoides* Cav. | Ch suffr | Mexico | Subtropical | | Cultivated |
| Lamiaceae | *Salvia fulgens* Cav. | Ch suffr | Mexico | Tropical | | Cultivated |
| Lamiaceae | *Salvia leucantha* Cav. | Ch frut | C America | Tropical | Neophyte | Naturalized alien |
| Lamiaceae | *Salvia microphylla* Kunth | Ch suffr | C America | Subtropical | Neophyte | Casual alien |
| Lamiaceae | *Salvia officinalis* L. | Ch suffr | C-S Europe | Temperate | | Native |
| Lamiaceae | *Salvia rosmarinus* Spenn. | NP | Mediterranean | Temperate | | Native |
| Lamiaceae | *Salvia splendens* Sellow ex Nees | Ch frut | Brazil | Tropical | Neophyte | Casual alien |
| Viburnaceae | *Sambucus nigra* L. | P caesp, P scap | Europe, Caucasus | Temperate | | Native |

**Table S1.** List of the Sicilian ornamental taxa per Families, Growth Form (according to [41, 42]), Geographical Origin (derived from [41]), Biome Origin (according to [41]), Resident Time and Status (Native/Alien) in Italy (derived both from [43,44]). New records from Sicily are reported with an asterisk.

| FAMILIES | TAXA | GROWTH FORM | GEOGRAPHICAL ORIGIN | BIOME ORIGIN | RESIDENT TIME | STATUS IN ITALY |
|---|---|---|---|---|---|---|
| Asteraceae | *Santolina chamaecyparissus* L. | NP | S-E Europe | Temperate | Neophyte | Naturalized alien |
| Asteraceae | *Santolina rosmarinifolia* L. | P caesp | S-W Europe | Temperate | | Cultivated |
| Araliaceae | *Schefflera digitata* J.R.Forst. & G.Forst. | P caesp, P scap | New Zealand | Subtropical | | Cultivated |
| Anacardiaceae | *Schinus molle* L. | P scap | S America | Subtropical | Neophyte | Naturalized alien |
| Anacardiaceae | *Schinus terebinthifolia* Raddi* | P scap | S America | Tropical | Neophyte | Casual alien |
| Cactaceae | *Schlumbergera opuntioides* (Loefgr. & Dusén) D.R.Hunt | NP succ | Brazil | Tropical | | Cultivated |
| Anacardiaceae | *Searsia gerrardii* (Engl.) Moffett | P caesp | S Africa | Subtropical | | Cultivated |
| Anacardiaceae | *Searsia lancea* (L.f.) F.A.Barkley | P scap | S Africa | Shrubland | | Cultivated |
| Anacardiaceae | *Searsia pendulina* (Jacq.) Moffett | P caesp, P scap | S Africa | Shrubland | | Cultivated |
| Anacardiaceae | *Searsia pentaphylla* (Jacq.) F.A.Barkley ex Moffett | P scap | N-W Africa, Sicily, Israel | Subtropical | | Native |
| Anacardiaceae | *Searsia tripartita* (Ucria) Moffett | P caesp, P scap | Sicily, N Africa | Shrubland | | Native |
| Anacardiaceae | *Searsia undulata* (Jacq.) T.S.Yi, A.J.Mill. & J.Wen | P caesp | S Africa | Shrubland | | Cultivated |
| Crassulaceae | *Sedum adolphi* Raym.-Hamet | Ch succ | Mexico | Subtropical | Neophyte | Casual alien |
| Cactaceae | *Selenicereus anthonyanus* (Alexander) D.R.Hunt | P succ | Mexico | Tropical | | Cultivated |
| Cactaceae | *Selenicereus grandiflorus* (L.) Britton & Rose | P succ | C America | Tropical | | Cultivated |
| Cactaceae | *Selenicereus undatus* (Haw.) D.R.Hunt | P succ | C America | Tropical | Neophyte | Naturalized alien |
| Asteraceae | *Senecio angulatus* L.f. | P lian | Cape Province | Subtropical | Neophyte | Invasive alien |
| Asteraceae | *Senecio macroglossus* DC. | H rept | S Africa | Subtropical | | Cultivated |
| Fabaceae | *Senna artemisioides* (Gaudich. ex DC.) Randell | P scap | Australia | Shrubland | | Cultivated |
| Fabaceae | *Senna bicapsularis* (L.) Roxb. | P scap | S America | Tropical | | Cultivated |
| Fabaceae | *Senna surattensis* (Burm.f.) H.S.Irwin & Barneby | P caesp, P scap | S Asia | Tropical | | Cultivated |
| Cupressaceae | *Sequoia sempervirens* (D.Don) Endl. | P scap | Oregon, California, | Temperate | Neophyte | Naturalized alien |
| Fabaceae | *Sesbania punicea* (Cav.) Benth.* | P caesp, P scap | S America | Subtropical | Neophyte | Invasive alien |
| Sapotaceae | *Sideroxylon inerme* L. | P caesp, P scap | S Africa | Tropical | | Cultivated |
| Cactaceae | *Soehrensia spachiana* (Lem.) Schlumpb. | P succ | S America | Shrubland | Neophyte | Casual alien |
| Solanaceae | *Solandra maxima* (Moc. & Sessé ex Dunal) P.S.Green | P lian | C-S America | Tropical | Neophyte | Casual alien |
| Solanaceae | *Solanum amygdalifolium* Steud.* | P lian | S America | Subtropical | | Cultivated |
| Solanaceae | *Solanum laxum* Spreng. | P lian | S America | Subtropical | | Cultivated |
| Solanaceae | *Solanum pseudocapsicum* L. | NP | S America | Tropical | Neophyte | Naturalized alien |
| Solanaceae | *Solanum seaforthianum* Andrews | NP | S America | Tropical | Neophyte | Naturalized alien |
| Solanaceae | *Solanum wendlandii* Hook.f.* | P lian | C-S America | Tropical | | Cultivated |
| Asteraceae | *Solidago canadensis* L. | H scap | N America | Temperate | Neophyte | Invasive alien |
| Rosaceae | *Sorbus aucuparia* L. | P scap | Europe, N-C Asia | Temperate | | Native |

**Table S1.** List of the Sicilian ornamental taxa per Families, Growth Form (according to [41, 42]), Geographical Origin (derived from [41]), Biome Origin (according to [41]), Resident Time and Status (Native/Alien) in Italy (derived both from [43,44]). New records from Sicily are reported with an asterisk.

| FAMILIES | TAXA | GROWTH FORM | GEOGRAPHICAL ORIGIN | BIOME ORIGIN | RESIDENT TIME | STATUS IN ITALY |
|---|---|---|---|---|---|---|
| Malvaceae | *Sparrmannia africana* L.f. | P caesp, P scap | Cape Province | Subtropical | | Cultivated |
| Fabaceae | *Spartium junceum* L. | P caesp | Mediterranean | Temperate | | Native |
| Cyatheaceae | *Sphaeropteris cooperi* (Hook. ex F.Muell.) R.M.Tryon | G rhiz | Australia | Subtropical | | Cultivated |
| Rosaceae | *Spiraea ×vanhouttei* (Briot) Carrière | P caesp | Artificial hybrid | Temperate | Neophyte | Casual alien |
| Rosaceae | *Spiraea cantoniensis* Lour. | P caesp | China | Temperate | Neophyte | Casual alien |
| Rosaceae | *Spiraea chamaedryfolia* L. | P caesp | S-E Europe, C-N Asia | Temperate | | Native |
| Rosaceae | *Spiraea trilobata* L. | P caesp | C-N Asia | Temperate | | Cultivated |
| Apocynaceae | *Stapelia grandiflora* Masson | NP succ | S Africa | Shrubland | | Cultivated |
| Apocynaceae | *Stephanotis floribunda* Jacques | P lian | Madagascar | Tropical | | Cultivated |
| Strelitziaceae | *Strelitzia alba* (L.f.) Skeels | P scap | S Africa | Subtropical | | Cultivated |
| Strelitziaceae | *Strelitzia juncea* Andrews | G rhiz | Cape Province | Subtropical | | Cultivated |
| Strelitzaceae | *Strelitzia reginae* Banks | G rhiz | Cape Province | Subtropical | | Cultivated |
| Fabaceae | *Styphnolobium japonicum* (L.) Schott | P scap | China | Temperate | Neophyte | Casual alien |
| Styracaceae | *Styrax officinalis* L. | P caesp, P scap | S-E Europe | Temperate | | Native |
| Arecaceae | *Syagrus romanzoffiana* (Cham.) Glassman | P scap | S America | Tropical | Neophyte | Casual alien |
| Caprifoliaceae | *Symphoricarpos albus* (L.) S.F.Blake* | P caesp | N America, Mexico | Temperate | Neophyte | Naturalized alien |
| Asteraceae | *Symphyotrichum ericoides* (L.) G.L.Nesom | H scap | N-C America | Temperate | Neophyte | Casual alien |
| Oleaceae | *Syringa vulgaris* L. | P caesp, P scap | S-E Europe | Temperate | Neophyte | Naturalized alien |
| Myrtaceae | *Syzygium cumini* (L.) Skeels | P scap | Australia | Tropical | | Cultivated |
| Asteraceae | *Tagetes erecta* L. | T scap | C America | Subtropical | Neophyte | Casual alien |
| Talinaceae | *Talinum paniculatum* (Jacq.) Gaertn.* | Ch succ | C-S America | Tropical | Neophyte | Casual alien |
| Tamaricaceae | *Tamarix africana* Poir. | P caesp, P scap | W Mediterranean | Subtropical | | Native |
| Tamaricaceae | *Tamarix arborea* (Sieber ex Ehrenb.) Bunge | P scap | N Africa, S Europe | Shrubland | | Native |
| Tamaricaceae | *Tamarix canariensis* Willd. | P caesp | Canary Is. | Subtropical | | Cultivated |
| Tamaricaceae | *Tamarix gallica* L. | P caesp | S-W Europe, N-W Africa | Temperate | | Native |
| Tamaricaceae | *Tamarix meyeri* Boiss. | P caesp, P scap | C-W Asia | Temperate | Neophyte | Naturalized alien |
| Tamaricaceae | *Tamarix parviflora* DC. | P caesp, P scap | S-E Europa, Turkey, Israel | Subtropical | Neophyte | Naturalized alien |
| Tamaricaceae | *Tamarix rosea* Bunge | P caesp | E Mediterranean | Temperate | Neophyte | Naturalized alien |
| Tamaricaceae | *Tamarix tetragyna* Ehrenb. | P caesp, P scap | E Mediterranean, Arabian Peninsula | Subtropical | | Native/Cryptogenic |
| Fabaceae | *Tara spinosa* (Molina) Britton & Rose | P scap | C-S America | Tropical | Neophyte | Casual alien |
| Taxaceae | *Taxus baccata* L. | P scap | Europe, N-W Africa, Caucasus | Temperate | | Native |
| Taxaceae | *Taxus baccata* L. 'Fastigiata'* | P scap | Horticultural | Temperate | | Cultivated |
| Taxaceae | *Taxus baccata* L. 'Fastigiata Aurea'* | P scap | Horticultural | Temperate | | Cultivated |

**Table S1.** List of the Sicilian ornamental taxa per Families, Growth Form (according to [41, 42]), Geographical Origin (derived from [41]), Biome Origin (according to [41]), Resident Time and Status (Native/Alien) in Italy (derived both from [43,44]). New records from Sicily are reported with an asterisk.

| FAMILIES | TAXA | GROWTH FORM | GEOGRAPHICAL ORIGIN | BIOME ORIGIN | RESIDENT TIME | STATUS IN ITALY |
|---|---|---|---|---|---|---|
| Taxaceae | *Taxus baccata* L. 'Variegata' | P scap | Horticultural | Temperate | | Cultivated |
| Bignoniaceae | *Tecoma stans* (L.) Juss. ex Kunth | P caesp | C-S America | Tropical | Neophyte | Casual alien |
| Bignoniaceae | *Tecomaria capensis* (Thunb.) Spach | P caesp | S Africa | Subtropical | Neophyte | Casual alien |
| Cupressaceae | *Tetraclinis articulata* (Vahl) Mast. | P scap | S-W Mediterranean | Subtropical | | Cultivated |
| Araliaceae | *Tetrapanax papyrifer* (Hook.) K.Koch | P caesp, P scap | China, Taiwan | Tropical | Neophyte | Casual alien |
| Vitaceae | *Tetrastigma harmandii* Planch. | P lian | S-E Asia | Tropical | | Cultivated |
| Lamiaceae | *Teucrium fruticans* L. | NP | Mediterranean | Subtropical | | Native |
| Cupressaceae | *Thuja occidentalis* L. | P scap | N-E America | Temperate | Neophyte | Casual alien |
| Cupressaceae | *Thuja plicata* Donn ex D.Don | P scap | N America | Temperate | Neophyte | Casual alien |
| Acanthaceae | *Thunbergia coccinea* Wall. ex D.Don | P lian | China | Tropical | | Cultivated |
| Acanthaceae | *Thunbergia grandiflora* Roxb. | P lian | S-E Asia | Tropical | | Cultivated |
| Malvaceae | *Tilia ×europaea* L. | P scap | Europe | Temperate | | Cultivated |
| Malvaceae | *Tilia americana* L. | P scap | N-C America | Temperate | Neophyte | Casual alien |
| Malvaceae | *Tilia cordata* Mill. | P scap | Europe, N-C Asia | Temperate | | Native |
| Malvaceae | *Tilia platyphyllos* Scop. | P scap | Europe, Caucasus | Temperate | | Native |
| Malvaceae | *Tilia tomentosa* Moench | P scap | C-S-E Europa, Turkey | Temperate | Neophyte | Naturalized alien |
| Fabaceae | *Tipuana tipu* (Benth.) Kuntze | P scap | S America | Subtropical | | Cultivated |
| Taxaceae | *Torreya nucifera* (L.) Siebold & Zucc. | P scap | Korea, Japan | Temperate | | Cultivated |
| Apocynaceae | *Trachelospermum jasminoides* (Lindl.) Lem. | P lian | E Asia | Subtropical | Neophyte | Casual alien |
| Arecaceae | *Trachycarpus fortunei* (Hook.) H.Wendl. | P scap | E Asia | Temperate | Neophyte | Invasive alien |
| Commelinaceae | *Tradescantia fluminensis* Vell. | G rhiz | S America | Tropical | Neophyte | Invasive alien |
| Commelinaceae | *Tradescantia pallida* (Rose) D.R.Hunt | G rhiz | Mexico | Tropical | Neophyte | Casual alien |
| Commelinaceae | *Tradescantia pallida* (Rose) D.R.Hunt 'Purpurea'* | G rhiz | Horticultural | Tropical | | Cultivated |
| Commelinaceae | *Tradescantia sillamontana* Matuda | G rhiz | Mexico | Subtropical | Neophyte | Casual alien |
| Tropaeolaceae | *Tropaeolum majus* L. | T rept | Peru | Subtropical | Neophyte | Invasive alien |
| Amaryllidaceae | *Tulbaghia violacea* Harv. | G bulb | Cape Province | Subtropical | Neophyte | Casual alien |
| Liliaceae | *Tulipa agenensis* Redouté | G bulb | E Mediterranean | Subtropical | Neophyte | Naturalized alien |
| Liliaceae | *Tulipa gesneriana* L. | G bulb | Turkey | Temperate | Neophyte | Casual alien |
| Ulmaceae | *Ulmus glabra* Huds. | P scap | Europe, N-W Asia | Temperate | | Native |
| Ulmaceae | *Ulmus minor* subsp. *canescens* Bartolucci & Galasso | P scap | C-W-E Mediterranean | Temperate | | Native |
| Ulmaceae | *Ulmus parvifolia* Jacq. | P scap | E Asia | Temperate | | Cultivated |
| Fabaceae | *Vachellia farnesiana* (L.) Wight & Arn. | P scap | C-S America | Tropical | Neophyte | Casual alien |
| Fabaceae | *Vachellia karroo* (Hayne) Banfi & Galasso | P scap | S Africa | Shrubland | Neophyte | Invasive alien |

**Table S1.** List of the Sicilian ornamental taxa per Families, Growth Form (according to [41, 42]), Geographical Origin (derived from [41]), Biome Origin (according to [41]), Resident Time and Status (Native/Alien) in Italy (derived both from [43,44]). New records from Sicily are reported with an asterisk.

| FAMILIES | TAXA | GROWTH FORM | GEOGRAPHICAL ORIGIN | BIOME ORIGIN | RESIDENT TIME | STATUS IN ITALY |
|---|---|---|---|---|---|---|
| Fabaceae | *Vachellia nilotica* subsp. *tomentosa* (Benth.) Kyal. & Boatwr. | P scap | N-C Africa, India | Shrubland | | Cultivated |
| Fabaceae | *Vachellia tortilis* (Forssk.) Galasso & Banfi | P scap | Africa | Shrubland | | Cultivated |
| Caprifoliaceae | *Valeriana rubra* L. | Ch suffr | C-W Mediterranean | Temperate | | Cultivated |
| Rutaceae | *Vepris lanceolata* (Lam.) G.Don | P caesp, P scap | C-S Africa | Tropical | | Cultivated |
| Plantaginaceae | *Veronica salicifolia* G.Forst. | P caesp | New Zealand | Temperate | | Cultivated |
| Viburnaceae | *Viburnum odoratissimum* Ker Gawl. | P caesp | S-E Asia | Subtropical | | Cultivated |
| Viburnaceae | *Viburnum opulus* L. | P caesp | Europe, N-C Asia, N-W Africa | Temperate | | Native |
| Viburnaceae | *Viburnum rhytidophyllum* Hemsl. | P caesp, P scap | China | Temperate | Neophyte | Casual alien |
| Viburnaceae | *Viburnum tinus* L. | P caesp | Mediterranean | Subtropical | | Native |
| Viburnaceae | *Viburnum tinus* var. *lucidum* (Mill.) Aiton | P caesp | S Europe | Subtropical | | Native |
| Apocynaceae | *Vinca major* L. | Ch rept | S Europe, Caucasus | Temperate | | Native |
| Apocynaceae | *Vinca major* var. *variegata* Loudon | Ch rept | Horticultural | Temperate | | Cultivated |
| Violaceae | *Viola odorata* L. | H ros | Europe, N-W Africa, W Asia | Temperate | | Native |
| Lamiaceae | *Vitex agnus-castus* L. | P caesp, P scap | Mediterranean, C-S Asia | Subtropical | | Native |
| Vitaceae | *Vitis vinifera* L. | P lian | C-S-E Europe, Caucasus | Temperate | | Native |
| Arecaceae | *Washingtonia filifera* (T.Moore & Mast.) H.Wendl. ex de Bary | P scap | California, Arizona, Mexico | Shrubland | Neophyte | Naturalized alien |
| Arecaceae | *Washingtonia filifera* var. *robusta* (H.Wendl.) Parish | P scap | Mexico | Shrubland | Neophyte | Naturalized alien |
| Caprifoliaceae | *Weigela florida* (Bunge) A.DC.* | P caesp | C Asia | Temperate | Neophyte | Casual alien |
| Caprifoliaceae | *Weigela florida* (Bunge) A.DC. 'Variegata'* | P caesp | Horticultural | Temperate | | Cultivated |
| Rosaceae | *Weniomeles bodinieri* (H.Lév.) B.B.Liu | P scap | China, Vietnam | Temperate | | Cultivated |
| Boraginaceae | *Wigandia urens* (Ruiz & Pav.) Kunth | P scap | C-S America | Tropical | Neophyte | Naturalized alien |
| Fabaceae | *Wisteria floribunda* (Willd.) DC. | P lian | Japan | Temperate | Neophyte | Casual alien |
| Fabaceae | *Wisteria sinensis* (Sims) DC. | P lian | China | Temperate | Neophyte | Naturalized alien |
| Sapindaceae | *Xanthoceras sorbifolium* Bunge | P caesp, P scap | China, Korea | Temperate | | Cultivated |
| Asphodelaceae | *Xanthorrhoea preissii* Endl. | P caesp | W Australia | Subtropical | | Cultivated |
| Asparagaceae | *Yucca aloifolia* L. | P caesp | USA, C America | Tropical | Neophyte | Naturalized alien |
| Asparagaceae | *Yucca aloifolia* var. *variegata* Rothsch. | P caesp | Horticultural | Tropical | Neophyte | Naturalized alien |
| Asparagaceae | *Yucca faxoniana* (Trel.) Sarg. | P scap | Mexico, Texas | Shrubland | | Cultivated |
| Asparagaceae | *Yucca filifera* Chabaud | P scap | Mexico | Shrubland | | Cultivated |
| Asparagaceae | *Yucca gigantea* Lem. | P caesp | C America | Tropical | Neophyte | Casual alien |
| Asparagaceae | *Yucca gloriosa* L. | P caesp | USA | Temperate | Neophyte | Invasive alien |
| Asparagaceae | *Yucca rostrata* Engelm. ex Trel.* | P caesp, P scap | Mexico | Shrubland | | Cultivated |
| Zamiaceae | *Zamia furfuracea* L.f. ex Aiton* | NP | Mexico | Tropical | | Cultivated |

**Table S1.** List of the Sicilian ornamental taxa per Families, Growth Form (according to [41, 42]), Geographical Origin (derived from [41]), Biome Origin (according to [41]), Resident Time and Status (Native/Alien) in Italy (derived both from [43,44]). New records from Sicily are reported with an asterisk.

| FAMILIES | TAXA | GROWTH FORM | GEOGRAPHICAL ORIGIN | BIOME ORIGIN | RESIDENT TIME | STATUS IN ITALY |
|---|---|---|---|---|---|---|
| Araceae | *Zantedeschia aethiopica* (L.) Spreng. | G rhiz | S Africa | Tropical | Neophyte | Invasive alien |
| Fabaceae | *Zapoteca portoricensis* (Jacq.) H.M.Hern. | P scap | C-S America | Tropical | | Cultivated |
| Asteraceae | *Zinnia elegans* Jacq. | T scap | C America | Tropical | Neophyte | Casual alien |
| Rhamnaceae | *Ziziphus lotus* (L.) Lam. | P caesp | N Africa, S Europe | Subtropical | | Native |
